# A stable systemic infection of methicillin-resistant *Staphylococcus aureus* (MRSA) in cynomolgus macaques produces extended window for therapeutic intervention

**DOI:** 10.3389/fmicb.2025.1601381

**Published:** 2025-07-14

**Authors:** Adrienne J. Gamblin, Max C. Peterson, Samantha S. Simon, Tatyana Orekov, Erica Penn, Gabriella Shumyatsky, Grishma Patel, Amanda Womer, Helal Kabir, Darnell Bushby, Jeanne Muench, Karen Feliciano, Fabian Paz, Brad Finneyfrock, Anthony Cook, Swagata Kar

**Affiliations:** BIOQUAL, Inc., Rockville, MD, United States

**Keywords:** *Staphylococcus aureus*, MRSA, cynomolgus macaque, nonhuman primate, *S. aureus* bacteremia, bacterial infection model, sepsis

## Abstract

**Introduction:**

*Staphylococcus aureus* is a common gram-positive commensal that, upon entering the bloodstream, can cause devastating illness and death within hours or days. Methicillin-resistant *S. aureus* (MRSA) infections, now a leading cause of bloodstream infections worldwide, pose significant challenges due to their rapid progression, high mortality rates, and limited therapeutic options. While there are prevalent small animal models of experimental MRSA infection, there has been minimal development of larger mammalian models capable of recapitulating clinical aspects of human systemic MRSA infection.

**Methods:**

Following a pilot study to determine the optimal dose and route to establish systemic MRSA infection, we challenged six cynomolgus macaques with 10^9^ colony-forming units MRSA (lineage USA300) via intravenous (IV) route. Animals were monitored closely up to 8 days for physiological, immunological, and cellular endpoints. Histopathology was performed on tissues collected 2 and 8 days after infection.

**Results:**

An IV dose of 10^9^ CFU MRSA USA300 in cynomolgus macaques produced bacteremia resulting from multifocal invasive infections, elevated markers of systemic inflammation, as well as weight loss, fever, and hemodynamic changes consistent with bloodstream infection. Hematological analyses demonstrated neutrophilic leukocytosis, lymphocytopenia, monocytosis, and mild thrombocytopenia. We observed a robust cytokine response, including TNF-α, IL-6, G-CSF, and IL-1RA, peaking 6 h post-infection. Flow cytometry immunophenotyping revealed dynamic shifts in circulating monocyte subpopulations, and histopathological analysis demonstrated multi-organ damage with significant findings in the kidneys, heart, liver, and lungs. By 8 days post-infection, moderate to severe myocardial, renal, and hepatic dysfunction were evident, supported by changes in clinical chemistry biomarkers. None of the animals required euthanasia before the scheduled date of termination.

**Discussion:**

In this study, we establish a non-human primate model of systemic MRSA infection that allows for the characterization of MRSA pathogenesis and evaluation of therapeutics over a period of days rather than hours. This model successfully recapitulates key aspects of human MRSA bloodstream infections, providing a valuable platform for evaluating therapeutic interventions and understanding disease mechanisms.

## Introduction

*Staphylococcus aureus* is a gram-positive bacterium commonly found in the skin and nasal microbiota of 20–30% of the population (Kluytmans et al., 1997; Wertheim et al., 2005). While typically associated with localized and self-limiting skin infections, it has emerged as a leading cause of bloodstream infections worldwide, significantly impacting morbidity, mortality, and healthcare costs (Diekema et al., 2019). The severity of *S. aureus* bacteremia stems from its potential to cause life-threatening complications, including pneumonia, infective endocarditis, meningitis, metastatic infections (e.g., osteomyelitis, abscesses), and septic shock (Tong et al., 2015). Between 10 and 30% of affected patients succumb to complications, a mortality rate exceeding that of AIDS, tuberculosis, and viral hepatitis (van Hal et al., 2012). The clinical burden of *S. aureus* is magnified by its propensity for accumulating antimicrobial resistance mutations, including those against vancomycin and methicillin (McGuinness et al., 2017; Turner et al., 2019). Circulating strains of methicillin-resistant *S. aureus* (MRSA) are of greatest clinical concern, as the gene that is responsible for methicillin resistance also confers broad resistance to most other β-lactam antibiotics. Since 2013, the United States Centers for Disease Control and Prevention (CDC) has listed MRSA as a serious threat to human health (CDC, 2013, 2019). In meta-analyses of hospital cases, MRSA bloodstream infections have been found to carry a risk of mortality two to five times higher than that of non-resistant strains (Whitby et al., 2001; Cosgrove et al., 2003). In the United States alone, MRSA was responsible for nearly 200,000 bloodstream infections and over 19,000 associated deaths in a single year (Kourtis et al., 2019). MRSA strains are broadly categorized by their epidemiological origin, which reflects how the infections are acquired. Healthcare-associated MRSA (HA-MRSA) strains, which are typically contracted in medical settings (i.e., hospitals, nursing homes, and dialysis clinics) can differ considerably in their clinical manifestations, drug resistance profiles, virulence factors, and transmissibility compared to community-associated MRSA (CA-MRSA) strains, which emerge outside of healthcare settings and infect otherwise healthy persons. However, the traditional distinctions between HA-MRSA and CA-MRSA have begun to collapse, as CA-MRSA transmission has recently been reported in hospital settings (Popovich et al., 2017). The CA-MRSA USA300 lineage has become a leading cause of hospital-associated bloodstream infections in the United States (Tosas Auguet et al., 2016; Souli et al., 2019; Seybold et al., 2006; Tenover et al., 2012; Tickler et al., 2017). MRSA’s genetic agility in the global context of antimicrobial resistance puts a time limit on the utility of current treatment options, primarily vancomycin (a β-lactam antibiotic) and daptomycin (a lipopeptide). Resistance to both have already been observed in MRSA isolates (Watkins et al., 2019). In order to develop and evaluate new classes of therapeutics against MRSA, a variety of *in vivo* models will be needed. Systemic *S. aureus* infections have largely been studied in rodents and rabbits, owing to their small size, low cost, and in the case of mice, genetic tractability for the creation of humanized strains that recapitulate some aspects of the human immune system (Marra, 2020; Hung et al., 2022). Non-human primate (NHP) models, which often bridge the gap between early preclinical studies and human trials, have been left critically underdeveloped in MRSA research. To date, the utilization of NHPs to investigate systemic *S. aureus* infection/sepsis has been limited to a single study, in which baboons were challenged with a lethal dose (4 × 10^10^ CFU/kg) that caused death on the order of hours (Hinshaw et al., 1992). This short therapeutic window leaves little time to assess drugs in development and limits researchers’ ability to study the pathogenesis of *S. aureus* bloodstream infections. Additionally, the ethical concerns of inducing such extreme illness in these animals warrant extraordinary restraint and care in developing NHP models of invasive bacterial infections. An ideal animal model of systemic MRSA infection should recapitulate the hypotension, fever, and elevated white blood cell counts seen in human patients, activate similar cytokine and chemokine pathways, and result in multiple organ dysfunction (Salas et al., 2017; Chen et al., 2019). At the same time, the model must be both economically reasonable and stable enough to allow extended observation and collection of biological samples. While mice and other small animals meet some of these criteria for systemic bacterial infection models, considerable limitations exist (Stortz et al., 2017). Hemodynamic monitoring is technically challenging in small animals, and low total blood volume limits serial collections for cellular and molecular analyses. Additionally, murine innate immune responses to experimental bacterial infection differ significantly from human immune responses in clinical settings, and vary across mouse strains and challenge methods (Chen et al., 2014; Kim et al., 2014; Mrochen et al., 2020). Every MRSA vaccine candidate that was developed using murine models has failed in clinical trials, underscoring the need for evolutionarily closer animal models of MRSA infection (Clegg et al., 2021; Miller et al., 2020). The baboon (*Papio ursinus* or *cynocephalus*) model of bacterial sepsis, in which animals are intravenously challenged with gram-negative *E. coli*, has served for decades as the only well-established option for investigation of bacterial blood infection pathogenesis and assessment of therapeutic interventions for bacteremia in an NHP model (Chen et al., 2019). However, recent pressures on NHP stocks due to COVID-19 pandemic-related supply chain disruption and a changing regulatory environment have made the cynomolgus macaque an attractive alternative to the baboon, which has become prohibitively difficult to obtain for most investigators (National Academies of Sciences, 2023). Additionally, the unique threat of MRSA, a gram-positive organism with virulence mechanisms distinct from the gram-negative *E. coli*, merits its own model for translational preclinical work (Feezor et al., 2003). As such, we selected the cynomolgus macaque for this preliminary investigation of MRSA bloodstream infection in an NHP model. In this study, we establish a tractable NHP model of MRSA bacteremia and systemic infection, providing a platform for studying MRSA pathogenesis and evaluating therapeutic interventions. Based on the results from a dose-finding pilot study, we hypothesized that an IV dose of 10^9^ colony-forming units (CFU) of CA-MRSA USA300 could establish a sublethal infection in cynomolgus macaques for over 48 h. At 2 days post-infection, all six macaques challenged at this dose survived, and the three animals not designated for terminal collection at 2 dpi survived through 8 dpi. Bacteremia driven by multifocal invasive infections rather than the initial bacterial bolus was detected in four of the six animals, and evidence of established tissue infections was observed in five of six animals. This model enables a comprehensive characterization of physiological, cellular, and molecular responses to systemic MRSA infection in cynomolgus macaques, which we hope will encourage translational research bridging the gap between murine studies and clinical trials.

## Materials and methods

### Animals

The pilot study population consisted of five young adult female cynomolgus macaques (*Macaca fascicularis*) (age, 5.1 ± 0.9 years; weight 3.17 ± 0.23 kg). The core study population consisted of six (1 female and 5 male) young adult cynomolgus macaques (age 4.1 ± 0.1 years; weight 6.59 ± 1.02 kg). All animals in the pilot and core study were housed in a facility accredited by AAALAC (The Association for Assessment and Accreditation of Laboratory Animal Care, International) in accordance with the Animal Welfare Act and Regulations, the *Guide for the Care and Use of Laboratory Animals* (8th edition), and Public Health Service Policy. All animal use described in this study was approved by BIOQUAL’s Institutional Animal Care and Use Committee (IACUC), which includes the provision of standard veterinary care. The pilot study was performed under IACUC Protocol #18-025P; the core study was performed under IACUC Protocol #24-003. The studies were also conducted in accordance with AALAS (The American Association for Laboratory Animal Science) position statements on Humane Care and Use of Laboratory Animals and Alleviating Pain and Distress in Laboratory Animals. Prior to study entry, animals were screened for simian T-cell leukemia virus (STLV), simian immunodeficiency virus (SIV), and Macacine herpesvirus 1 (herpes B virus) via serology, and simian retrovirus (SRV) types 1 through 5 via serology followed by PCR analysis; all animals enrolled in the pilot and core studies tested negative. Additionally, all animals enrolled in the studies were determined to be negative for tuberculosis through entry/quarantine screening and biannual intradermal testing. Additional screening specific to these studies included nasal swab culture for presence of *Acinetobacter* spp., *Corynebacterium* spp., *Klebsiella pneumoniae, Trichosporon* spp., *and Staphylococcus aureus* bacteria. In the core study, four animals were excluded from an initial 10 animals (five male, five female) for presence of *S. aureus* in nasal swabs, resulting in six animals (five male, one female) cleared for study. All study animals were found healthy and fit for study through review of clinical history, physical examination, and review of serum chemistry and hematology. Before any observations or procedures requiring handling, animals were sedated with an intramuscular injection of ketamine HCl (10–25 mg/kg). Following MRSA challenge during the core study, cages were not changed until scheduled euthanasia at 2 and 8 dpi to further minimize the risk of cross-contamination. All animals were euthanized in accordance with AVMA (American Veterinary Medical Association) guidelines by barbiturate overdose via intravenous (IV) catheter at the saphenous vein, on the opposite leg used for bacterial challenge.

### Husbandry and housing

Animals were housed in an indoor facility ventilated with 100% fresh air at a range of 10–15 air changes hourly and maintained at 72 ± 2°F (21.5 ± 1°C), 30–0% relative humidity, and a 12 h:12 h light:dark photoperiod. Animals were housed under ABSL-2 conditions. To mitigate the risk for unintended bacterial transmission to neighboring animals, prior to MRSA inoculation, NHPs completed a minimum 3-day acclimation period to biocontainment enclosures with isolator blower filter units. NHPs were housed in visual and auditory contact with conspecifics and participated in the institution’s behavioral and environmental enrichment program. This program includes monitoring by an animal behaviorist, cohousing of animals in successful pairs or groups prior to inoculation, uninterrupted access to various toys and forage materials, and auditory enrichment. Animals were fed a commercial NHP diet (LabDiet Monkey Diet 5038, Purina) in amounts appropriate for weight and growth stage once daily, fresh fruits and vegetables once daily, and were provided *ad libitum* access to municipal tap water.

### Bacteria strain and source

The USA300 MRSA stock (USA 300-0114) used for bacterial challenge in this study was purchased from BEI Resources (BEI, Cat. No. NR-46070). This USA300 strain (also referred to as NRS384) was isolated from a patient’s wound in Mississippi, United States, and is a community-acquired MRSA (CA-MRSA). Strain USA300-0114 was deposited to BEI as resistant to erythromycin and tetracycline; positive for *mec* (subtype IV); *pvl*^+^; MLST sequence type (ST) 8; eGenomic *spa* type 1, eGenomic *spa* repeats YHGFMBQBLO; Ridom *spa* type t008; *agr* grp I. The certificate of analysis from the vendor confirmed antibiotic resistance to erythromycin, tetracycline, oxacillin, and benzylpenicillin. According to the BEI data sheet provided, this strain matched the virulent specifications of MRSA, including catalase and coagulase positivity and β-hemolysis, and identity was confirmed by the vendor via a fluorescence-based method (VITEK 2 Compact) measuring the rate of bacterial growth and biochemical reactions, which were compared to a database of cataloged bacterial species.

### Bacterial stock preparation

MRSA USA300 bacterial stock (BEI, Cat. No. NR-46070) was streaked on selective differential BBL™ CHROMagar^®^ MRSA II plates (BD, Cat. No. 215228) and incubated overnight at 37°C. Isolated MRSA colonies were picked based on color and morphology, and cultured (one colony per tube) in 10 mL tryptic soy broth (TSB) media (Millipore, Cat. No. 146317) overnight in a shaker at 240 rpm at 37°C. Subcultures were generated in TSB in 250 mL polycarbonate flasks (Corning, Cat. No. 430183) and incubated on a shaker at 240 rpm until the OD_600_ reached 0.8–0.9 (stationary phase OD_600_ = 0.05). As recommended by the BBL™ CHROMagar^®^ MRSA II plate manufacturer’s instructions, a Staphaurex latex agglutination test (ThermoFisher Scientific, Cat. No. R30859901) was performed to confirm that the in-house stock generated from the BEI commercial stock possessed coagulase and/or Protein A, which differentiates *S. aureus* from other staphylococci. Flask contents were transferred to 50 mL tubes and centrifuged at 5,000 × g at 4°C to pellet the bacterial cells. The cells were washed 1x with phosphate-buffered saline (PBS), resuspended in 10 mL freezing media (10% glycerol in PBS), and then frozen in aliquots of 300 μL at −80°C. To confirm the titer of these stocks, frozen aliquots were thawed, serially diluted in PBS, and plated on Trypticase Soy Agar (TSA) plates supplemented with 5% sheep blood (Fisher Scientific, Cat. No. R01200). Plates were incubated overnight at 37°C followed by bacterial enumeration expressed in CFU/mL. The stock titer (10 × 10^10^ CFU/mL) was verified by quantitative bacteriology of three aliquots performed by two separate analysts; the coefficient of variation (CV) across replicates was 23%.

### Inoculum preparation

The bacterial stock for the pilot study was freshly grown on the day of challenge, then diluted and washed as described below. For the core study, frozen bacterial stock vials were thawed at room temperature in a water bath and homogenized by pipetting until a uniform solution was achieved. The thawed stock was washed 3x in 1 mL PBS and centrifuged at 5,000 × g at 4°C for 2 min for each spin. After the final wash, the pellets were resuspended in 300 μL 1x PBS and serially diluted to prepare challenge inoculum at the intended dose. After the challenge procedures, remaining challenge inoculum was further diluted and plated in duplicate on TSA plates supplemented with 5% sheep blood (Fisher Scientific, Cat. No. R01200) to verify the titer. The plates were incubated overnight at 37°C and colonies were enumerated to determine CFU/mL. The mean titer of the challenge dose verification replicates was 1.6 × 10^9^ CFU/mL.

### Pilot study design

The pilot study population was challenged with 10^7^–10^10^ colony-forming units (CFU) of MRSA USA300 via IV or intraperitoneal (IP) route. Each animal was challenged via a single dose/route combination: 10^10^ CFU IV, 10^9^ CFU IV, 10^8^ CFU IV, 10^10^ CFU IP, or 10^7^ CFU IP. Physical exams (including recording of body weight, body temperature, blood pressure, and clinical observations) were performed frequently between 0 and 48 h post-infection (hpi), and once daily thereafter until study termination at 7 days post-infection (dpi). Blood samples were collected during physical exams for bacteriology and hematology analyses. At necropsy, heart, lung, liver, and spleen were collected from select animals for bacteriology.

### Core study design

The core study population was challenged with 10^9^ CFU of MRSA USA300 via IV route. Physical exams (including recording of body weight, body temperature, blood pressure, and clinical observations) were performed frequently between 0 and 48 hpi, and once daily subsequently until study termination at 2 dpi (*n* = 3 animals) and 8 dpi (*n* = 3 animals). Blood samples were collected during physical exams for bacteriology, hematology, clinical chemistry, immunoassay, and flow cytometry analyses. At necropsy, the following tissues were collected for histopathological assessment: heart, lung, liver, left and right kidney, spleen, lymph nodes (axillary & inguinal), injection site (skin, saphenous vein region), skin of the saphenous vein region of the opposite leg, small intestine, and large intestine.

### Blood pressure measurement and mean arterial pressure estimation

Blood pressure was measured using a non-invasive indirect blood pressure sphygmomanometer (SurgiVet^®^ V60046). An appropriately sized cuff was placed on the animal’s arm and the average of three readings was calculated and documented for each physical examination. Systolic pressure (SP) and diastolic pressure (DP) readings were used to estimate mean arterial pressure (MAP) with the widely used Gauer equation, where MAP = DP + 1/3(SP–DP) (Gauer, 1960).

### Bacteriology

As a measure of bacteremia, whole blood serially diluted in PBS was plated, in triplicate, on TSA plates supplemented with 5% sheep blood (Fisher Scientific, Cat. No. R01200) and incubated overnight at 37°C. The CFU/mL of whole blood was determined by averaging the colony counts from three replicates of 1–2 plating dilutions.

### Enzyme-linked immunosorbent assay (ELISA) for the detection of C-reactive protein

A kit-based enzyme-linked immunosorbent assay (ELISA) (LSBio, Cat. No. LS-F32737) was used to detect C-reactive protein (CRP) in the serum samples collected from the NHPs assigned to the study. The assay was performed following manufacturer’s provided instructions, including appropriate quality controls and standards.

### Hematology, blood morphology, and serum biochemistry analyses

Whole blood samples collected in ethylenediaminetetraacetic acid (EDTA) tubes and sera collected in serum separation tubes (SST) were sent to IDEXX BioAnalytics (Westbrook, ME) for complete blood count (CBC), blood morphology (blood smear), and serum biochemistry (CHEM) analyses (IDEXX Custom Research Profile 60863). Whole blood was assayed for total white blood cell (WBC) counts, total red blood cell (RBC) counts, total hemoglobin (HGB), hematocrit (HCT), mean corpuscular volume (MCV), mean corpuscular hemoglobin (MCH), mean corpuscular hemoglobin concentration (MCHC), WBC differentials (e.g., absolute and% neutrophils, lymphocytes, monocytes, eosinophils, and basophils), and platelet (PLT) counts. A whole blood morphology analysis (blood smear) was performed to assess for polychromasia (presence of immature red blood cells), anisocytosis (variation in red blood cell size), poikilocytosis (abnormal red blood cell shape), hemolysis (destruction of red blood cells), Heinz bodies (inclusions of denatured hemoglobin in red blood cells), blood clots, as wells as observations of platelet estimates, platelet clumping and platelet abnormalities. Serum was assayed for concentrations of alkaline phosphatase (ALP), alanine transaminase (ALT), aspartate transaminase (AST), creatine kinase (CK), albumin (ALB), total protein (TP), globulin (GLOB), total bilirubin (TBIL), conjugated and unconjugated bilirubin, blood urea nitrogen (BUN), creatinine (CRE), total cholesterol (TCHO), glucose (GLU), calcium (Ca), inorganic phosphorus (IP), bicarbonate (HCO_3_), chloride (Cl), potassium (K), and sodium (Na). ALB/GLOB, BUN/CRE, and Na/K ratios were also determined from serum in the same IDEXX panel.

### Cytokine profiling

Circulating cytokine, chemokine, and growth factor concentrations were evaluated using a ProcartaPlex^®^ NHP Cytokine/Chemokine/Growth Factor Panel (ThermoFisher Scientific; Cat. No. EPX320-40045-901) for 37 soluble analytes: GM-CSF, IFN-γ, IL-1β, IL-10, IL-12p70, IL-13, IL-17α, IL-18, IL-2, IL-23, IL-4, IL-5, IL-6, TNF-α, BLC, EOTAXIN, IL-8, IP-10, I-TAC, MCP-1, MIG, MIP-1α, MIP-1β, SDF-1α, CD40-LIGAND, G-CSF/CSF-3, IFN-α, IL-1RA, IL-15, IL-7, BDNF, bNGF, FGF-2, PDGF-BB, SCF, VEGF-A, and VEGF-D. This 37-plex immunoassay, based on Luminex^®^ xMAP^®^ technology, was performed on serum samples according to the manufacturer’s instructions, which included appropriate standards and quality controls. Samples were run in duplicate, and data was acquired on a Luminex 200™ instrument system.

### Flow cytometry for immunophenotyping

Whole blood samples (100 μL/well) were washed 1x in PBS (500 x g for 5 min at 2–8°C) and resuspended in Human TruStain FcX™ (Biolegend, Cat. No. 422302) prepared 1:20 in FBS Stain Buffer (BD Biosciences, Cat. No. 554656). Resuspended cells were Fc-blocked for 10 min, followed by a 30 min surface antibody staining with the following panel: BV605 Mouse Anti-NHP CD45 (Clone D058-1283, BD Biosciences, Cat. No. 564098), APC-Cy™7 Mouse Anti-Human CD3 (Clone SP34-2, BD Biosciences, Cat. No. 557757), PE Mouse Anti-Human CD4 (Clone L200, BD Biosciences, Cat. No. 550630), BV711 Mouse Anti-Human CD8 (Clone RPA-T8, BD Biosciences, Cat. No. 563677), PE-Cy™5 Mouse Anti-Human CD20 (Clone 2H7, BD Biosciences, Cat. No. 555624), Brilliant Violet 510™ Mouse Anti-Human CD16 (Clone 3G8, BioLegend, Cat. No. 302048), Alexa Fluor^®^ 700 Mouse Anti-Human CD14 (Clone M5E2, BD Biosciences, Cat. No. 557923), APC Mouse Anti-Human CD11b (Clone ICRF44, BioLegend, Cat. No. 301310), FITC Mouse Anti-Human CD66abce (Clone TET2, Miltenyi Biotec, Cat. No. 130-116-522), Brilliant Violet 570™ Mouse Anti-Human HLA-DR (Clone L243, BioLegend, Cat. No. 307638), Brilliant Violet 650™ Mouse Anti-Human CD11c (Clone Bu15, BioLegend, Cat. No. 337238), BV786 Mouse Anti-Human CD123 (Clone 7G3, BD Biosciences, Cat No. 564196), PE/Dazzle™ 594 Mouse Anti-Human CD56 (Clone HCD56, BioLegend, Cat. No. 318348). Samples were depleted of red blood cells and fixed using 1x FACS Lysis Buffer (BD Biosciences, Cat. No. 349202) for 20 min, washed twice in FBS Stain Buffer, and then resuspended in FBS Stain Buffer for acquisition. Sample acquisition was performed using a Cytek^®^ Aurora instrument (Cytek Biosciences) and analyzed with FlowJo™ software v.10 (BD Biosciences). After initial gating using forward scatter (FSC-A and FSC-H) and side scatter (SSC-A) to identify singlets with normal morphology (aka “live gate”), leukocytes (granulocytes, monocytes, and lymphocytes) were distinguished by CD45 expression and SSC-A. Unstimulated and stimulated neutrophils were distinguished by CD16 and CD11b expression. In the lymphocyte population, CD3 and CD20 expression was used to distinguish T cells, B cells, and a population containing NK cells; NK cells within this CD3^–^CD20^–^ population were identified by CD8 expression. The monocyte population was further characterized into classical (CD14^+^CD16^*int*^), intermediate (CD14^+^CD16^+^), and non-classical (CD14^*lo*^CD16^+^) monocytes. Plasmacytoid dendritic cells (pDCs) were distinguished with hierarchical gating using side scatter (SSC-A), CD56 expression, CD123 expression, and HLA-DR expression.

### Tissue collection and histopathology

Tissue samples were fixed in formalin and shipped to IDEXX BioAnalytics (Columbia, MO) for histopathologic assessments. Submitted tissues were trimmed and paraffin embedded, blocked, sectioned at 5 μm, mounted on slides, and stained with hematoxylin and eosin (H&E) for histopathologic scoring and evaluation by a board-certified veterinary pathologist. Serial sections were also obtained and stained with hematoxylin/Gram stain to evaluate for the presence of staphylococcal bacteria. International Harmonization of Nomenclature and Diagnostic (INHAND) Criteria standards were used as the basis of evaluation (Colman et al., 2021), with reference to published incidental findings in the cynomolgus macaque (Sato et al., 2012). Microscopic changes were graded according to severity utilizing a standard grading system whereby 0 = no significant change, 1 = minimal, 2 = mild, 3 = moderate, and 4 = severe. Use of numerical grades creates a mechanism to calculate total lesion scores, which were used to assess prevalence and severity of tissue changes among samples.

### Statistical analysis

Statistical analyses were performed using mixed-effects models for repeated measures, which employ the restricted maximum likelihood (REML) method. The Geisser-Greenhouse method was used to correct violations of sphericity in the data. In each analysis, a Dunnett’s multiple comparisons test was performed to compare the mean of each post-challenge time point with the mean of the baseline time point. In the text, any referenced *p*-values from multiple comparisons tests will include the *p* value in parentheses, followed by the comparison. For Luminex^®^ data, values reported out of range (OOR <) were substituted with the lower limit of quantification (LLOQ) for that analyte. All statistical analyses were performed using GraphPad Prism versions 10.1.0 through 10.5.0 (GraphPad Software, San Diego, CA).

## Results

### Dose and route determination pilot study in cynomolgus macaques

**FIGURE 1:**
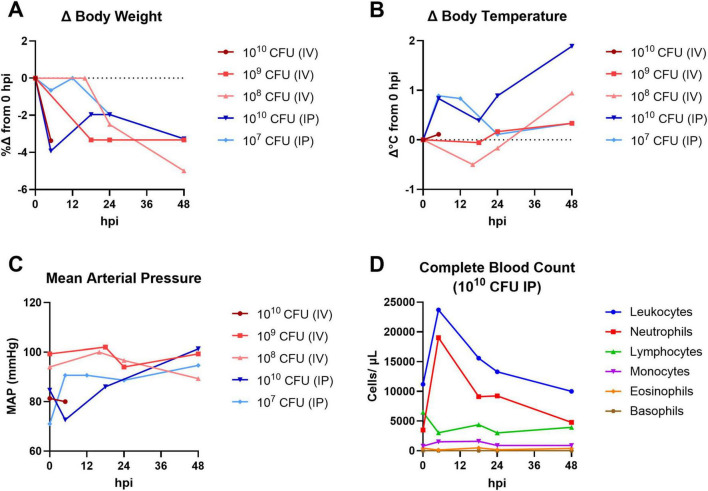
Pilot dose-finding study for systemic NHP MRSA infection model. Cynomolgus macaques were challenged with 10^10^–10^7^ CFU MRSA via IV or IP route and monitored for up to 7 days. Physical exams and blood collections were performed frequently between 0 and 48 hpi. **(A)** Percent change (%Δ) in body weight compared to baseline (0 hpi). **(B)** Change in rectal body temperature compared to baseline (0 hpi). **(C)** Mean arterial pressure (MAP) calculated from systolic and diastolic cuff readings. **(A**–**C)** The pilot animal that received a 10^10^ CFU dose via IV route (dark red) succumbed to infection between 5 and 18 hpi. **(D)** Absolute white blood cell counts from CBC hematology analysis of the pilot animal challenged with 10^10^ CFU MRSA via IP route.

To determine the optimal bacterial challenge dose and route for the characterization of systemic MRSA infection in the cynomolgus macaque, a pilot study (Figure 1) was conducted assessing a range of bacterial challenge doses (10^10^–10^7^ CFU) administered via IV or IP injection route. Animals were given physical exams and sampled for blood frequently between 0 and 48 hpi and were observed for general wellness until termination of the study at 7 dpi. The animal challenged with 10^10^ CFU via IV route succumbed to infection between 5 and 18 hpi. All other animals survived beyond the 48-h intensive observation period and were euthanized 7 dpi. Rapid body weight loss (Figure 1A) and spikes in body temperature (Figure 1B) were observed in most MRSA-infected animals within 48 h of infection; the severity and speed of onset of these changes appeared dose-responsive within groups that were infected via the same route. Estimation of mean arterial pressure (MAP) from systolic and diastolic pressure cuff readings did not reveal any major hemodynamic changes in the first 48 h of infection (Figure 1C). A complete blood count (CBC) analysis was performed on the pilot animal challenged with 10^10^ CFU MRSA via IP route; a sharp spike in total white blood cells (leukocytosis), particularly elevated neutrophils, was observed at 5 hpi (Figure 1D). CBC was also performed on whole blood collected from the pilot animal challenged with 10^10^ CFU MRSA via IV route; circulating total leukocytes and neutrophils were also elevated in this animal at the sole post-challenge time point prior to this animal’s death (data not shown).

To determine if MRSA challenge dose/route combinations in the pilot animals resulted in bacteremia, whole blood was collected and plated for semi-quantitative bacteriology at 0, 5, 18, 24, 48, and 96 hpi (Table 1). Bacterial colonies were detected in whole blood of the animals challenged with 10^10^ and 10^9^ CFU via IV route; none of the animals challenged via IP route had MRSA bacteria detectable in the blood. Homogenized tissues harvested from the animal that succumbed to infection early in the pilot study (10^10^ CFU IV) were also evaluated by bacteriology; heart, liver, lung, and spleen from this animal tested positive for MRSA bacteria. While both the 10^9^ and 10^8^ CFU IV challenge doses resulted in body weight loss (Figure 1A) and body temperature increases (Figure 1B), of the animals that survived to 7 dpi, only the NHP challenged with 10^9^ CFU via IV route demonstrated a systemic infection detectable by bacteriology (Table 1). Based on the results of the pilot study, we selected a challenge dose of 10^9^ CFU via IV route for our subsequent core study.

**TABLE 1:** MRSA bacteremia in pilot animal whole blood.

Dose and route	5 hpi	18 hpi	24 hpi	48 hpi	96 hpi
10^10^ CFU IV	**+**	**++**	n/a	n/a	n/a
10^9^ CFU IV	−	**++**	**+**	−	−
10^8^ CFU IV	−	−	−	−	−
10^10^ CFU IP	−	−	−	−	−
10^7^ CFU IP	−	−	−	−	−

Five cynomolgus macaques were challenged with 10^10^–10^7^ CFU MRSA via IV or IP route and sampled for blood at 5, 18, 24, 48, and 96 hpi; one animal was challenged per dose/route combination. Bacteremia was evaluated semi-quantitatively by plating diluted whole blood on TSA plates and comparing colony numbers and/or colony density when bacterial colonies were too numerous to count. A “−” indicates the absence of bacterial colonies, a “+” indicates an easily enumerated number of colonies, and a “++” indicates large numbers of colonies that were difficult to enumerate due to density.

### IV challenge of cynomolgus macaques with 10^9^ CFU MRSA recapitulates clinical aspects of bacterial blood infection

**FIGURE 2:**
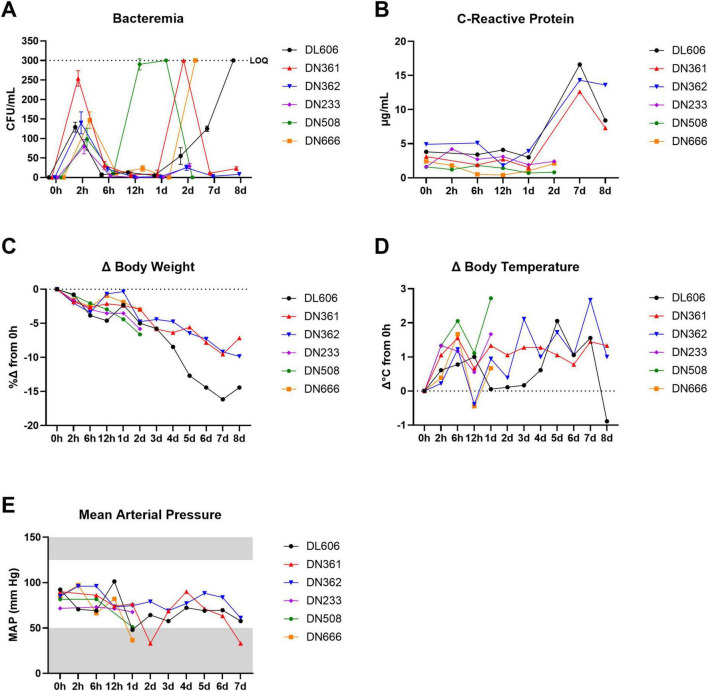
IV challenge of NHPs with 10^9^ CFU MRSA produces bacteremia, elevated CRP, and clinical signs of systemic infection. Six cynomolgus macaques were infected with 10^9^ CFU MRSA via IV route. Three of the animals were monitored for 2 dpi and then euthanized; the remaining three animals were monitored up to 8 dpi and then euthanized. **(A)** Bacteremia was assessed via semi-quantitative bacteriology of whole blood. The upper limit of quantification (LOQ) for this bacteriological assay was 300 CFU/mL (black dotted line). Data are shown as the mean ± SD of two replicate TSA plates. Data are interleaved for clarity. **(B)** Concentrations of CRP in serum determined via CRP-ELISA. **(C)** Percent changes (%Δ) in body weight are shown calculated based on baseline body weight recorded at 0 hpi. **(D)** Changes (Δ) in body temperatures are shown calculated on baseline body temperatures recorded rectally at 0 hpi. **(E)** Mean arterial pressure (MAP) was estimated from systolic and diastolic blood pressure data recorded during physical exams. Gray areas indicate values outside the normal clinical ranges for cynomolgus macaques.

To confirm that the dose and route identified in our pilot study produces a sublethal systemic infection, we challenged six cynomolgus macaques with 10^9^ CFU MRSA via IV route (Figure 2). Three animals (DN233, DN508, and DN666) were euthanized at 2 dpi for gross necropsy and histopathological assessment; the remaining animals survived to study termination at 8 dpi. Blood samples were assessed for bacteremia and C-reactive protein, and animals were monitored for body weight, body temperature, blood pressure, and clinical signs of disease throughout the study.

In this core study, a short initial burst of bacteremia was detectable in all animals at 2 hpi, likely resulting from the initial bolus of MRSA bacteria administered via IV route (Figure 2A). This initial bacteremia waned between 2 and 6 hpi as the innate immune system cleared the initial bacterial challenge bolus. Animal DN508 exhibited the earliest onset of bacteremia after this initial clearance phase, with a dramatic progression of bacteremia between 6 and 12 hpi that extended through 1 dpi. Two other animals (DN361 and DN666) reached peak bacteremia at 2 dpi, and animal DL606 presented with peak bacteremia at 8 dpi. Low levels of bacteremia were observed in animal DN362 and animal DN233 at 2 dpi. All six animals presented with detectable bacteremia at some point after the initial clearance phase.

To assess the systemic inflammatory response following MRSA infection, serum C-reactive protein (CRP) was evaluated by standard ELISA (Figure 2B). Despite the high inter-individual variation in the timing of bacteremia peaks, we found that CRP levels in sera remained near baseline in all animals until at least 2 dpi. Between 2 and 7 dpi, CRP levels jumped from < 5 μg/mL to > 12 μg/mL and peaked at 7 dpi.

We observed sharp declines in body weight from 2 to 6 hpi, with a brief recovery toward baseline in some animals between 6 and 12 hpi. After 1 dpi, body weights steadily declined until study termination (Figure 2C). A subclinical febrile peak of 1.4 ± 0.5°C was observed shortly after the initial bacteremia peak at 6 hpi (Figure 2D). Body temperatures briefly recovered toward baseline at 12 hpi, before generally increasing until 7–8 dpi. In animals that were not euthanized at 2 dpi, mean body temperatures remained elevated at least 7 dpi, with animal DL606 and animal DN362 exhibiting body temperatures at or above the clinical threshold for fever (39.5°C). Within the first 24 h of infection, we observed declines in estimated mean arterial pressure (MAP) > 15 mmHg in all animals except DN233 (Figure 2E). This animal exhibited the lowest bacterial load at 2 hpi, and was euthanized before robust bacteremia was detected. The lowest MAP recorded was 33 mmHg, in animal DN361, coinciding with that animal’s peak bacteremia at 2 dpi.

### MRSA IV challenge produces robust shifts in circulating white blood cell populations and red blood cell abnormalities consistent with systemic MRSA infection

**FIGURE 3:**
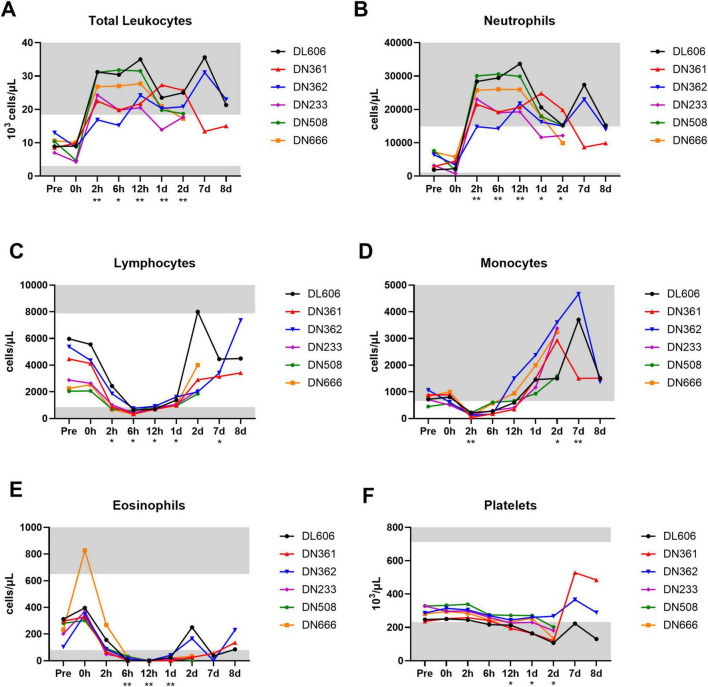
Changes in circulating white blood cell subtypes following IV MRSA challenge. Complete blood count (CBC) analyses were performed on an IDEXX analyzer for absolute **(A)** total white blood cells (leukocytes), **(B)** neutrophils, **(C)** lymphocytes, **(D)** monocytes, **(E)** eosinophils, and **(F)** platelets. **(A–F)** Statistical comparisons were performed using repeated-measures, mixed-effect analyses with Dunnett’s *post hoc* multiple comparisons test; *P < 0.05, **P < 0.01. Asterisks indicate *p*-values of multiple comparisons against the baseline “Pre” timepoint, and are shown below the x axis. Gray areas indicate values outside normal clinical ranges for cynomolgus macaques.

Complete blood count (CBC) hematology analyses were performed on whole blood samples from each animal throughout the core study. All six MRSA challenged animals exhibited total white blood cell (leukocyte) counts above the normal clinical range for cynomolgus macaques between 2 and 12 hpi, with many animals exhibiting leukocytosis persisting until termination (Figure 3A). This expansion of circulating leukocytes appears largely due to an increase in neutrophils (Figure 3B), though elevated monocytes (monocytosis) was also observed beginning ∼1 dpi and persisting until 8 dpi (Figure 3D). Lymphocytes (Figure 3C), monocytes (Figure 3D), and eosinophils (Figure 3E) decreased shortly after bacterial challenge, with at least partial recovery to baseline levels around 1 dpi. A gradual decrease in platelet counts (thrombocytopenia) was observed from 2 hpi to at least 2 dpi (Figure 3F).

In addition to CBC analyses, blood smears were performed on whole blood to assess for abnormalities in blood cell morphology (Table 2). MRSA-infected cynomolgus macaques exhibited immature red blood cells (polychromasia), variation in red blood cell size (anisocytosis) and shape (poikilocytosis), and breakdown of red blood cells (hemolysis). Platelet clumping was observed in a majority of blood smears at 2 and 8 dpi. Notably, fibrin strands were observed in 2/3 blood smears at 7 dpi, and blood clots were observed in animal DN508 at 2 dpi. The full hematology dataset is provided in Supplementary Data Sheet 1.

**TABLE 2:** Qualitative blood smear observations in MRSA-infected cynomolgus macaques.

Blood smear observation	Pre	0 hpi	2 hpi	6 hpi	12 hpi	1 dpi	2 dpi	7 dpi	8 dpi
Polychromasia	0/6	0/6	0/6	0/6	0/6	0/6	0/6	**1/3**	**1/3**
Anisocytosis	0/6	0/6	0/6	0/6	**1/6**	0/6	**4/6**	**2/3**	**3/3**
Poikilocytosis	**1/6**	0/6	0/6	0/6	0/6	0/6	**4/6**	**1/3**	0/3
Hemolysis	**3/6**	**5/6**	**3/6**	**4/6**	**6/6**	**5/6**	**3/6**	0/3	0/3
Heinz bodies	0/6	0/6	0/6	0/6	0/6	0/6	0/6	0/3	0/3
Platelet clumping	0/6	0/6	**1/6**	0/6	**1/6**	0/6	**4/6**	0/3	**2/3**
Giant platelets	0/6	**1/6**	**1/6**	0/6	0/6	0/6	0/6	0/3	0/3
Blood clots	0/6	0/6	0/6	0/6	0/6	0/6	**1/6**	0/3	0/3
Fibrin strands	0/6	0/6	0/6	0/6	0/6	0/6	0/6	**2/3**	0/3

Six cynomolgus macaques were challenged with 10^9^ CFU MRSA via IV route. Whole blood was collected in anticoagulant EDTA tubes and blood smears were performed as part of a custom hematological profile. Qualitative CBC parameters are summarized as follows: # of animals exhibiting observation/total # of animals. The number of animals displaying the associated blood smear observation are indicated in bold.

### MRSA IV challenge produces acute cytokine and chemokine response

**FIGURE 4:**
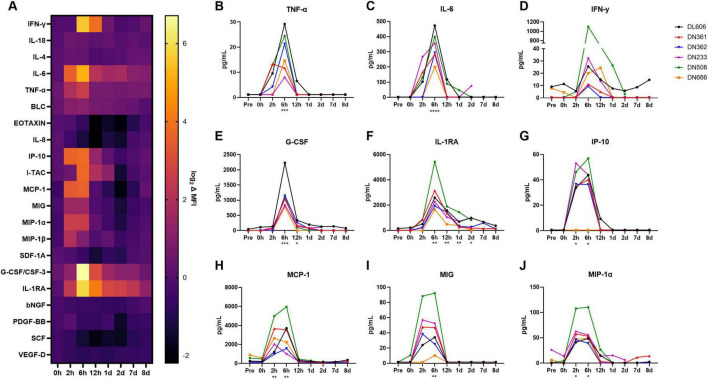
Host cytokine/chemokine immune response of cynomolgus macaques following IV infection with 10^9^ CFU MRSA. Concentrations of 37 cytokines, chemokines, and growth factors were characterized in cynomolgus macaque serum at baseline (Pre) through 8 dpi on the ProcartaPlex platform. **(A)** Heat map showing log-normalized change (Δ) in mean fluorescence intensity (MFI) of 21 analytes that were detected above the lower limit of detection in > 50% of animals. Serum concentrations of TNF-α **(B)**, IL-6 **(C)**, IFN-γ **(D)**, G-CSF **(E)**, IL-1RA **(F)**, IP-10 **(G)**, MCP-1 **(H)**, MIG **(I)**, and MIP-1α **(J)**, are shown in pg/mL. **(B–J)** Statistical comparisons were performed using repeated-measures, mixed-effect analyses with Dunnett’s *post hoc* multiple comparisons test; *P < 0.05, **P < 0.01, ***P < 0.001, and ****P < 0.0001. Asterisks indicate *p-*values of multiple comparisons against the baseline “Pre” timepoint and are shown below the x axis for clarity.

To assess whether a human-like innate immune response was induced in cynomolgus macaques following MRSA infection, we evaluated longitudinal serum samples for the expression of 37 cytokines and chemokines via Luminex^®^ immunoassay (Figure 4). Of these analytes, 21 (56.8%) were above the minimum level of detection in > 50% of animals and were included in the analysis (Supplementary Data Sheet 2).

We observed significant spikes in the expression of pro-inflammatory cytokines TNF-α (*p* = 0.0007) and IL-6 (*p* = 0.0012) around 6 hpi (Figures 4B,C). IFN-γ concentrations appeared elevated from baseline at 6 hpi, though this difference was not significant (*p* = 0.0518), as one animal (DN508) exhibited a spike many orders of magnitude larger than the other animals in the study (Figure 4D). Significantly increased expression of the anti-inflammatory cytokines G-CSF (*p* = 0.0121) and IL-1RA (*p* = 0.0007) was also observed around 6 hpi (Figures 4E,F). Of the chemokines, significant increases in expression were observed for IP-10 (*p* = 0.0036), MCP-1 (*p* < 0.0001), MIG (*p* = 0.0010), and MIP-1α (*p* = 0.0005) around 2–6 hpi (Figures 4G–J). MIP-1β also appeared elevated at 2 hpi; this difference was not significant (*p* = 0.0667) (Supplementary Image 1). By 1 dpi, cytokine and chemokine levels had largely returned to baseline. Notably, animals exhibiting later bacteremia peaks did not exhibit secondary spikes in these cytokines and chemokines.

### Immunophenotyping reveals major shifts in monocytic subpopulations and NK cells following MRSA infection

**FIGURE 5:**
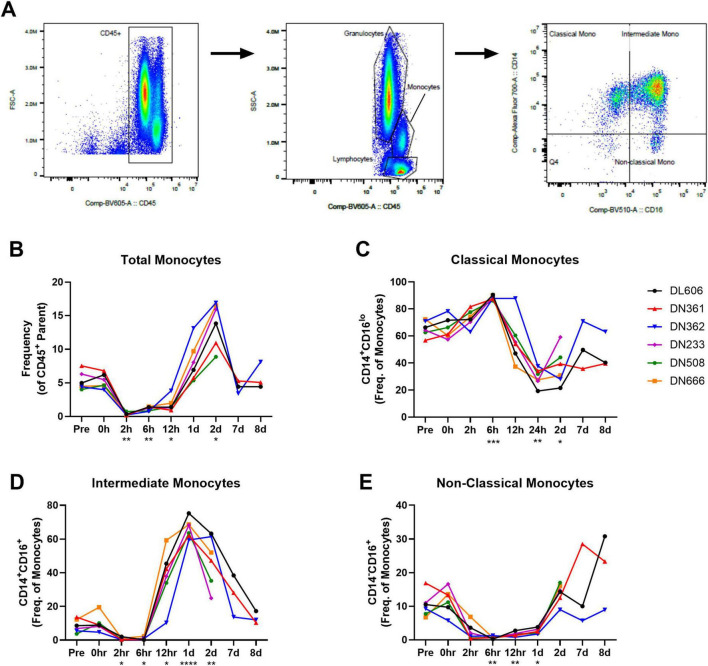
Monocyte subpopulations in whole blood of cynomolgus macaques infected with 10^9^ CFU MRSA via IV route. Immunophenotyping performed by flow cytometry on whole blood collected throughout the study reveals unique behavior of circulating monocyte populations. **(A)** Representative gating strategy for monocyte subpopulations. **(B)** Total monocytes shown as frequency of CD45 + parent population. **(C)** Classical monocytes shown as frequency of monocyte population. **(D)** Intermediate monocytes shown as frequency of monocyte population. **(E)** Non-classical monocytes shown as frequency of monocyte population. **(B–E)** Statistical comparisons were performed using repeated-measures, mixed-effect analyses with Dunnett’s *post hoc* multiple comparisons test; *P < 0.05, **P < 0.01, ***P < 0.001, ****P < 0.0001.

Given the observed increase in monocyte-associated chemokines IP-10 and MCP-1 shortly after infection, we examined the longitudinal frequency of monocyte subpopulations in circulation. Whole blood immunophenotyping was performed by flow cytometry. The gating strategy for monocytes (Figure 5A) was based on size, granularity, CD45 expression, and the expression of CD14 and CD16 resulting in the following subsets: CD14^+^CD16^lo^ classical, CD14^+^CD16^+^ intermediate, and CD14_*lo*_CD16^+^ non-classical. The full gating strategy for whole blood immunophenotyping is available in Supplementary Presentation 1.

Total monocytes in peripheral blood dropped immediately after MRSA challenge (*p* = 0.0020; Pre vs. 2 hpi), but recovered by 1 dpi and then increased well above pre-study baseline at 2 dpi (*p* = 0.0118; Pre vs. 2 dpi) (Figure 5B). Within the monocyte population, however, we observed an early increase in CD14^+^CD16_lo_ classical monocytes (*p* = 0.0005; Pre vs. 6 hpi) followed by a marked decline through 2 dpi (*p* = 0.0419; Pre vs. 2 dpi) (Figure 5C). In line with the behavior of the total monocyte population, an influx or expansion of intermediate CD14^+^CD16^+^ monocytes was seen after a brief post-challenge depletion, with intermediate CD14^+^CD16^+^ monocytes peaking 1 dpi (*p* < 0.0001; Pre vs. 1 dpi) (Figure 5D). Non-classical CD14_*lo*_CD16^+^ monocytes were also depleted from peripheral blood shortly after MRSA challenge, remaining in low numbers until around 2 dpi (Figure 5E).

As part of our flow cytometry immunophenotyping of whole blood, we also evaluated for T cells, B cells, and NK cells (Supplementary Image 2). While the numbers of CD4 + T cells, CD8 + T cells, and B cells remained relatively constant throughout the study, a significant depletion of NK cells from peripheral blood was observed almost immediately after MRSA challenge (*p* = 0.0011; Pre vs. 2 hpi), lasting through 12 hpi.

### Gross necropsy findings demonstrate systemic inflammation and establishment of organ infections as sequelae of MRSA bacteremia

Animals euthanized at 2 dpi showed consistent gross lesions at the kidneys and spleen (Table 3). Kidney lesions included generalized tissue pallor (DN508) and/or mild to moderate degrees of multifocal hemorrhagic nephritis (DN508 and DN233). Splenic findings consisted of moderate enlargement of the organ with rounded edges (DN508, DN233, and DN666). DN508 and DN666 displayed the additional finding of pinpoint necrotic foci on the serosal surface of the spleen.

**TABLE 3:** Gross lesions observed in spleen, kidney, liver, lung, and skin of MRSA-infected macaques at necropsy.

Animal ID	Necropsy day	Gross lesions
DN508	2 dpi	Moderate splenomegaly with rounded edges and pinpoint necrotic foci across the serosal surface. Generalized pale coloring of the surface of the left kidney.
DN233	2 dpi	Moderate splenomegaly with rounded edges. Moderate hemorrhage at the right kidney.
DN666	2 dpi	Moderate intra-abdominal fat. Mild-moderate splenomegaly with rounded edges and pinpoint necrotic foci across the serosal surface.
DL606	8 dpi	Moderate dehydration (delayed skin tent). Approximately 3 mm in diameter ruptured, superficial abscess between the shoulder blades with no active discharge at the time of exam. Generalized petechial hemorrhage throughout the lungs. Focal, necro-hemorrhagic lesion at the left kidney, multifocal hemorrhagic lesions at the right kidney. Moderate splenomegaly with rounded edges.
DN361	8 dpi	Moderate hepatomegaly, moderate splenomegaly, pale serosal surface of both right and left kidneys with moderate hemorrhage at the medulla. Mild-moderate gaseous distension throughout the intestinal tract.
DN362	8 dpi	Focal, pinpoint area of necrosis at the left kidney. Moderate splenomegaly.

Six cynomolgus macaques were challenged with 10^9^ CFU MRSA via IV route. Animals were euthanized and necropsied at 2 dpi (*n* = 3) and 8 dpi (*n* = 3).

As anticipated, animals euthanized at 8 dpi presented with more involved gross lesions than those animals assessed at 2 dpi (Table 3). DL606 displayed a superficial, dime-sized ruptured skin abscess between the shoulder blades 2 days prior to scheduled terminal collections, which was treated in-life with daily sterile water lavage. This same animal also displayed thoracic cavity lesions in the form of generalized petechial hemorrhage throughout the lung surface. All three animals (DN361, DN362, and DL606) in the 8 dpi necropsy group exhibited gross lesions at the kidney and spleen. The spleen was consistently found to be moderately enlarged with rounded edges. Kidney findings consisted of a focal, necro-hemorrhagic lesion, multifocal hemorrhagic lesions, and pale serosal surfaces (DN362 and DL606) with dark hemorrhage throughout the medulla (DN361). Animal DN361 displayed additional findings of moderate hepatomegaly.

### Cynomolgus macaques infected with 10^9^ CFU IV MRSA exhibit multiple organ damage within 2 days of infection

**FIGURE 6:**
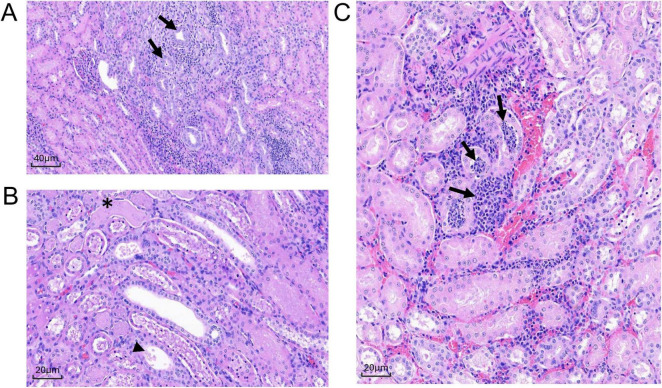
Histologic sections of MRSA-challenged cynomolgus macaque kidney harvested from DN508 and DN233 at 2 dpi. **(A)** H&E stain (200x) of multifocal tubular degeneration (arrows) with regeneration and inflammation. **(B)** H&E stain (400x) of tubular epithelial necrosis (asterisk) with eosinophilic granular or inflammatory cell casts (arrowhead). **(C)** H&E stain (400x) of peritubular or tubular neutrophilic inflammation (arrows).

**FIGURE 7:**
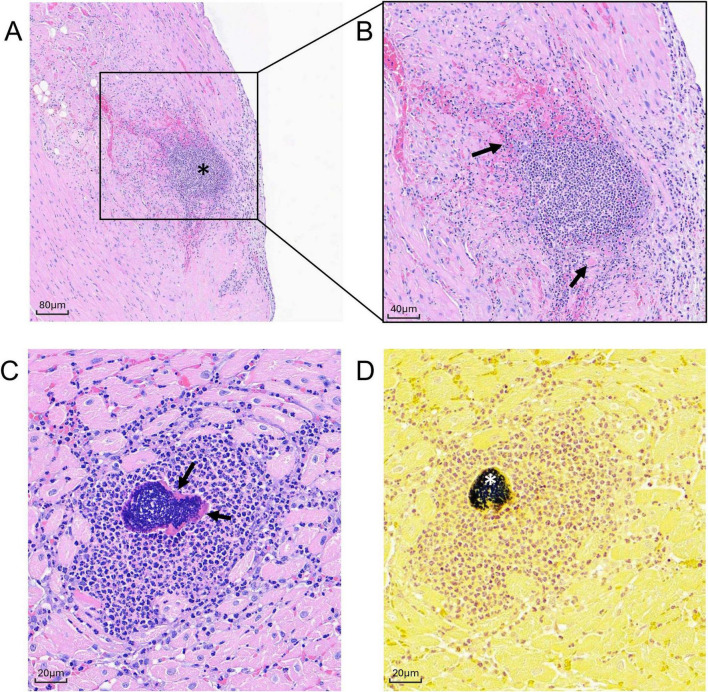
Histologic sections of MRSA-challenged cynomolgus macaque heart tissue harvested at 2 dpi. **(A,B)** H& E stain of cardiac section from animal DN508 showing moderate neutrophilic inflammation (asterisk) with coalescing area of myocardial necrosis (arrows) at **(A)** 100x, and **(B)** 200x. **(C,D)** Successive cardiac sections (400x) harvested from animal DN508 showing **(C)** H&E strain showing foci of neutrophilic inflammation and Splendore-Hoeppli structures (arrows) and **(D)** hematoxylin/Gram stain with moderate numbers of gram-positive cocci (asterisk).

Tissues from three cynomolgus macaques (DN233, DN508, and DN666) were examined by histological assessment for bacterial challenge-related effects present 2 dpi following IV administration with 10^9^ CFU MRSA. At this early time point, there were no microscopic findings in the lung, small intestine, large intestine, inguinal lymph node or opposite injection site. However, microscopic changes of minimal to moderate severity were present in the liver, kidney, heart, spleen, axillary lymph node and injection site at 2 dpi (Table 4). The most severe effects at this early time point were evident in the kidney (Figure 6) and heart (Figure 7).

**TABLE 4:** Individual histopathological findings in MRSA-challenged cynomolgus macaques at 2 dpi.

Tissue and histopathological finding	DN508	DN666	DN233	# Abnormal	Mean score
**Lung 2 dpi**					
Hemorrhage with necrosis, alveoli	0	0	0	0	0.0
Inflammation, mixed cell, alveolar/intravascular	0	0	0	0	0.0
Gram positive cocci present	0	0	0	0	0.0
Sum Scores	0	0	0	-	**0.0**
**Liver 2 dpi**					
Inflammation, neutrophilic and/or mixed cell, multifocal	1	1	2	3	1.3
Necrosis, hepatocellular, single cell, multifocal, acute	1	1	1	3	1.0
Hypertrophy/hyperplasia, Kupffer cell	1	1	1	3	1.0
Gram positive cocci present	0	0	0	0	0.0
Sum scores	3	3	4	–	**3.3**
**Kidney 2 dpi**					
Degeneration/necrosis, tubular, multifocal	1	0	1	2	0.7
Tubular casts, inflammatory cell	1	0	0	1	0.3
Inflammation, peritubular/tubular, neutrophilic	1	0	0	1	0.3
Infiltrate, lymphocytic, interstitial/peritubular	0	0	1	1	0.3
Microthrombus, acute with inflammation	0	0	1	1	0.3
Gram positive cocci present	0	0	0	0	0.0
Sum Scores	3	0	3	-	**2.0**
**Spleen 2 dpi**					
Cellularity increased, red pulp	2	0	1	2	1.0
Germinal Center, Acidophilic material	1	0	0	1	0.3
Infarction/necrosis, red pulp, regionally extensive	2	0	0	1	0.7
Gram positive cocci present	2	0	0	1	0.7
Sum Scores	7	0	1	–	**2.7**
**Axillary lymph node 2 dpi**					
Cellularity increased medullary sinus	1	0	0	1	0.3
Increased cellularity, histiocyte, medullary sinus, subcapsular sinus	1	0	0	1	0.3
Increased cellularity, lymphoid, germinal center	1	0	0	1	0.3
Gram positive cocci present	0	0	0	0	0.0
Sum scores	3	0	0	–	**1.0**
**Injection site (Skin) 2 dpi**					
Hemorrhage/edema, localized, dermis/subcutis/perivascular	1	0	2	2	0.3
Inflammation, mixed cell, dermal/subcutis, perivascular	1	0	2	2	0.3
Gram positive cocci present	0	0	0	0	0.0
Sum scores	2	0	4	–	**0.6**
**Heart 2 dpi**					
Inflammation, myocardial, neutrophilic, multifocal, acute	3	1	1	3	1.7
Degeneration/necrosis, myocardial, multifocal, acute	1	1	1	3	1.0
Inflammatory cell infiltrate, lymphocytic	1	0	0	1	0.3
Metaplasia, squamous, epicardial	0	0	1	1	0.3
Gram positive cocci present	2	0	0	1	0.7
Sum Scores	7	2	3	–	**4.0**

Tissue sections collected at necropsy (2 dpi) were stained with hematoxylin and eosin (H&E), and serial sections were stained with hematoxylin/Gram stain to evaluate the presence of bacteria. Microscopic changes were graded for severity using a standard grading system whereby 0 = no significant change, 1 = minimal, 2 = mild, 3 = moderate, and 4 = severe. International Harmonization of Nomenclature and Diagnostic (INHAND) Criteria standards were used as the basis of evaluation. Tissues without significant findings are not shown. The tissue sum of mean scores are indicated in bold.

In the kidney, microscopic changes at 2 dpi were minimal in severity and scattered throughout both kidneys in two animals (DN508 and DN233). Multifocal tubular degeneration with tubular epithelial necrosis was associated with minimal peritubular or tubular neutrophilic inflammation (Figure 6A) and the presence of eosinophilic granular or inflammatory cell casts (Figure 6B) were evident in both animals. A small microthrombus with neutrophilic inflammation was present in animal DN233 (Figure 6C). There were no discernible microscopic changes in the kidney of animal DN666. There were no discernible bacteria evident by H&E or Gram stain in the kidney of any animal at 2 dpi.

In the heart, microscopic changes of variable severity and incidence were evident in all three macaques at 2 dpi. Minimal to moderate multifocal neutrophilic inflammation was present throughout the heart (Figure 7). Moderate changes in DN508 were characterized by a large coalescing area of inflammation with myocardial necrosis (Figures 7A,B) with moderate numbers of gram-positive cocci in the central area (Figure 7D) and Splendore-Hoeppli structures (a hallmark of *S. aureus* infection) surrounding gram-positive organisms (Figure 7C). Minimal, multifocal acute myocardial degeneration and necrosis, characterized by randomly scattered necrotic or degenerating cardiomyocytes often associated with foci of neutrophilic inflammation were present in animal DN666 and animal DN233, though gram-positive microorganisms were not detected in the heart tissues of these animals at this time point (Table 4).

### Multi-organ dysfunction is widespread by 8 dpi in macaques infected with 10^9^ CFU IV MRSA

Tissues from three cynomolgus macaques (DN606, DN361, and DN362) were examined by histological assessment for bacterial challenge-related effects present 8 dpi following IV administration with 10^9^ CFU MRSA. Bacterial challenge-related findings were present in the kidney, heart, liver, lung, spleen, axillary lymph node, injection site and large intestine (Table 5). In most tissues, the severity and incidence of findings was higher than 2 dpi findings. There were no microscopic findings in the small intestine, inguinal lymph node, or opposite injection site at 8 dpi.

**TABLE 5:** Individual histopathological findings in MRSA-challenged cynomolgus macaques at 8 dpi.

Tissue and histopathological finding	DN361	DN362	DL606	# Abnormal	Mean score
**Lung 8 dpi**					
Hemorrhage with necrosis, alveoli	1	2	3	3	1.2
Inflammation, mixed cell, alveolar/intravascular	0	0	0	0	0.0
Gram positive cocci present	0	1	2	2	0.6
Sum scores	1	3	5	–	**1.8**
**Liver 8 dpi**					
Inflammation, neutrophilic and/or mixed cell, multifocal	2	1	1	3	1.3
Necrosis, hepatocellular, single cell, multifocal, acute	2	0	0	1	0.7
Hypertrophy/hyperplasia, Kupffer cell	1	1	1	3	1.0
Gram positive cocci present	0	0	0	0	0.0
Sum scores	5	2	2	–	**3.0**
**Kidney 8 dpi**					
Degeneration/necrosis, tubular, multifocal	2	2	3	3	2.3
Tubular casts, inflammatory cell	2	2	2	3	2.0
Inflammation, peritubular/tubular, neutrophilic	3	2	3	3	2.7
Infiltrate, lymphocytic, interstitial/peritubular	0	0	0	0	0.0
Microthrombus, acute with inflammation	0	0	4	1	1.3
Gram positive cocci present	0	0	3	1	1.0
Sum Scores	7	6	15	–	**8.3**
**Spleen 8 dpi**					
Cellularity increased, red pulp	1	2	2	3	1.7
Germinal Center, Acidophilic material	0	0	0	0	0.0
Infarction/necrosis, red pulp, regionally extensive	0	0	1	1	0.3
Gram positive cocci present	1	1	1	3	1.0
Sum Scores	2	3	4	-	**3.0**
**Axillary lymph node 8 dpi**					
Cellularity increased medullary sinus	0	0	0	0	0.0
Increased cellularity, histiocyte, medullary sinus, subcapsular sinus	0	1	1	2	0.7
Increased cellularity, lymphoid, germinal center	0	1	0	1	0.3
Gram positive cocci present	0	0	0	0	0.0
Sum scores	0	2	1	–	**1.0**
**Injection site (skin) 8 dpi**					
Hemorrhage/edema, localized, dermis/subcutis/perivascular	0	0	1	1	0.3
Inflammation, mixed cell, dermal/subcutis, perivascular	0	0	1	1	0.3
Gram positive cocci present	0	0	0	0	0.0
Sum Scores	0	0	3	–	**0.9**
**Heart 8 dpi**					
Inflammation, myocardial, neutrophilic, multifocal, acute	1	1	1	3	1.0
Degeneration/necrosis, myocardial, multifocal, acute	1	1	1	3	1.0
Inflammatory cell infiltrate, lymphocytic	0	1	1	2	0.7
Metaplasia, squamous, epicardial	0	0	0	0	0.0
Gram positive cocci present	0	0	1	1	0.3
Sum Scores	2	3	4	–	**3.0**
**Large intestine 8 dpi**					
Degeneration/regeneration, mucosal	2	0	0	1	0.7
Edema, submucosa	2	0	0	1	0.7
Inflammation, mixed cell	2	0	0	1	0.7
Sum Scores	6	0	0	–	**2.0**

Tissue sections collected at necropsy (8 dpi) were stained with H&E, and serial sections were stained with hematoxylin/Gram stain to evaluate the presence of bacteria. Microscopic changes were graded for severity using a standard grading system whereby 0 = no significant change, 1 = minimal, 2 = mild, 3 = moderate, and 4 = severe. International Harmonization of Nomenclature and Diagnostic (INHAND) Criteria standards were used as the basis of evaluation. Tissues without significant findings are not shown. The tissue sum of mean scores are indicated in bold.

**FIGURE 8:**
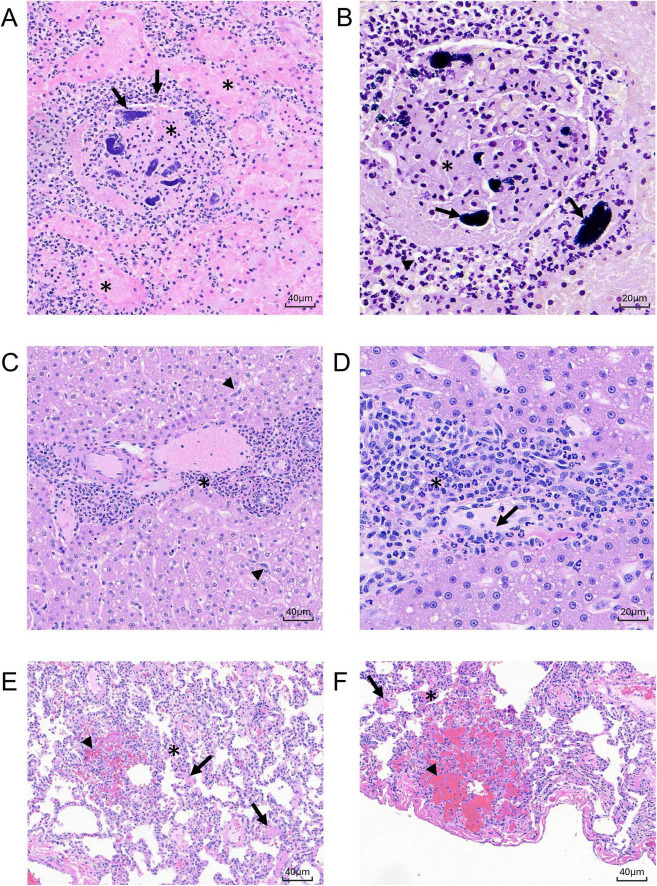
Histologic sections of MRSA-challenged cynomolgus macaque kidney, liver, and lung harvested at 8 dpi. **(A)** H&E stain (200 x) of kidney section from animal DL606 showing severe regional area of glomerular vascular thrombosis with renal parenchymal infarction/necrosis (asterisks), vascular wall necrosis, and fibrin deposition. **(B)** Hematoxylin/Gram stain (400x) of sequential kidney section from animal DL606 showing abundant neutrophilic inflammatory cells (arrowhead), and gram-positive cocci (arrows). **(C)** H&E stain (200x) of liver section from animal DN362 showing moderate neutrophilic or mixed cell inflammation with a primarily periportal distribution (asterisk), reactive hypertrophy and hyperplasia of sinusoidal Kupffer cells/macrophages, and expansion of the hepatic sinusoids with reactive appearing cells of histiocytic or endothelial origin (arrowheads). **(D)** H&E stain (400 x) of liver section from animal DN361 showing increased intravascular neutrophils and monocytic infiltrates with mild scattered single cell hepatocellular necrosis (arrow). **(E–F)** H& E stain of lung section from animal DN361 at 200x **(E)** and animal DL606 at 400x **(F)** showing minimal multifocal and regionally extensive areas of alveolar hemorrhage with alveolar septal necrosis (arrowheads) and numerous intravascular fibrin microthrombi (arrows). A mixed cell inflammation, consisting primarily of neutrophils and monocytes, expanded the alveolar septae; increased intravascular inflammatory cell infiltrate (asterisk) was observed.

Microscopic changes in the kidney at 8 dpi were present in all animals and consisted of mild to moderate multifocal tubular epithelial degeneration and necrosis with cellular tubular casts (Figures 8A,B). Associated with these changes was peritubular edema (DN606) and hemorrhage (DN361, DL606). In animal DN362, there was evidence of more chronic changes of tubular degeneration with tubular epithelial cell regeneration and peritubular fibrosis. The most severe changes in the kidney were present in animal DL606, where there was a focal area of vascular thrombosis with renal parenchymal infarction/necrosis, and vascular wall necrosis with fibrin deposition. Abundant numbers of gram-positive cocci were evident in the kidney both by H&E and Gram staining.

Microscopic changes in the liver were evident at 8 dpi in all three animals and were variable in both severity and incidence (Figures 8C,D). Minimal to moderate neutrophilic or mixed cell inflammation with a primarily periportal distribution was present in all three animals, with reactive hypertrophy and hyperplasia of sinusoidal Kupffer cells/macrophages. There was an increased number of intravascular neutrophils and monocytic infiltrates with mild scattered single cell hepatocellular necrosis, expansion of the hepatic sinusoids with reactive appearing cells, likely of histiocytic or endothelial origin. These changes were present adjacent to the portal areas, often extending to the limiting plate of the portal region and are consistent with immune activation of systemic inflammation. No microorganisms were identified in the liver by H&E or Gram stain.

Microscopic changes in the lung were present at 8 dpi in all animals and were variable in severity (Figures 8E,F). Minimal (DN361) to moderate (DL606) multifocal and regionally extensive areas of alveolar hemorrhage with alveolar septal necrosis and numerous intravascular fibrin microthrombi. Minimal to mild numbers of gram-positive microorganisms were present in tissue sections associated with these changes. A mixed cell inflammation (primarily neutrophils and monocytes) expanded the alveolar septae and there was an increased intravascular inflammatory cell infiltrate.

In the heart, microscopic changes were evident in all three animals at 8 dpi (Table 5). Minimal scattered foci of neutrophilic inflammation with acute cardio-myofiber coagulative necrosis and degeneration were present in all three animals. Associated with these changes was an increase in fibroblasts, indicating a more chronic or sub-chronic injury. In the spleen, bacterial challenge-related effects present 8 dpi were minimal to mild in severity and were similar to those present in animals 2 dpi (Table 5). Increased cellularity of the splenic red pulp was observed, primarily involving the splenic sinusoids with prominent reticuloendothelial hyperplasia and endothelial cell hypertrophy involving the splenic cords and sinuses. Animal DN508 had a mild localized splenic infarction (avascular necrosis) with vascular and parenchymal necrosis involving the sinusoidal red pulp. Minimal numbers of gram-positive cocci were present in the red pulp of all animals, and a regionally extensive area of necrosis/infarction was present in the red pulp of animal DL606. Minimal microscopic changes in the axillary lymph node were present in two animals (DN361 and DN362). Increased numbers of macrophage/histiocytes were evident in the medullary and subcapsular lymphoid sinuses (Table 5), though there were no discernible bacteria evident by H&E or Gram stain.

### Clinical blood chemistry markers support significant damage to liver, kidney, and heart tissues post infection

**FIGURE 9:**
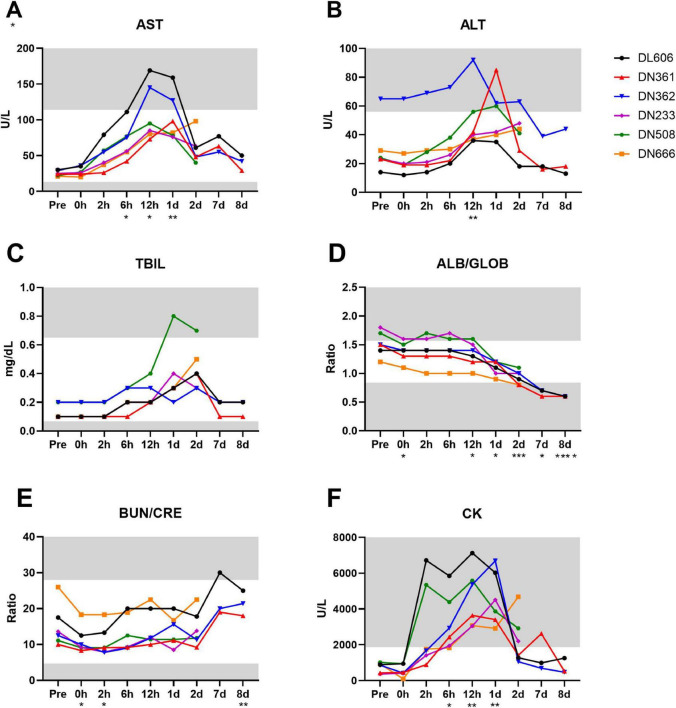
Clinical chemistry markers of liver, kidney, and other organ function are disrupted throughout MRSA infection of cynomolgus macaques. Analyte concentrations and ratios were determined by serum biochemistry panel on IDEXX platform. **(A)** Concentration of aspartate transferase (AST) in units per liter. **(B)** Concentration of alanine transaminase (ALT) in units per liter. **(C)** Concentration of total bilirubin (TBIL) in mg/dL. **(D)** Ratio of albumin to globulin (A/G) concentrations. **(E)** Ratio of blood urea nitrogen (BUN) to creatinine (CRE) concentrations. **(F)** Concentration of creatine kinase (CK) in units per liter **(A–F)** Statistical comparisons were performed using repeated-measures, mixed-effect analyses with Dunnett’s *post hoc* multiple comparisons test; *P < 0.05, **P < 0.01, ***P < 0.001, ****P < 0.0001. Asterisks indicate *p*-values of multiple comparisons against the baseline “Pre” timepoint and are shown below the x axis for clarity. Gray areas indicate values outside the normal clinical ranges for cynomolgus macaques.

There was notable serological evidence of liver damage in the study population between 6 and 24 hpi, with a significant spike in serum AST around 12 hpi (*p* = 0.0039) (Figure 9A). ALT, another core indicator of liver function, was significantly increased at 12 hpi compared to baseline (*p* = 0.0081; Pre vs. 12 hpi) (Figure 9B). Animals DN361 and DN362, which displayed the greatest peaks in serum ALT, also exhibited the most severe microscopic findings in the liver (Table 5). Total bilirubin (TBIL) was elevated above normal clinical range only in animal DN508, though small peaks were observed in several other animals at 1 and 2 dpi (Figure 9C). GGT levels remained largely unchanged throughout the study, except in animal DL606, which demonstrated elevated GGT at 8 dpi (Supplementary Image 3).

While there is considerable overlap between serological indicators of liver and kidney dysfunction, the ratios of circulating albumin to globulin (ALB/GLOB) and blood urea nitrogen to creatinine (BUN/CRE) are more closely associated with kidney disease or damage. We observed a significant downward trend in ALB/GLOB ratios among the study animals (*p* < 0.0001) with values dipping below normal clinical range around 2 dpi (Figure 9D). In addition, while the BUN/CRE levels remained mostly within normal limits throughout the study, they were significantly increased from baseline by 8 dpi (*p* = 0.0076; Pre vs. 8 dpi) (Figure 9E).

Elevated creatine kinase (CK) is an indicator of muscle injury and is associated with damage to the myocardium (Bodor, 2016). All animals displayed CK levels well above the normal clinical range by 12 hpi (Figure 9F). CK levels were significantly increased from baseline at 6 hpi (*p* = 0.0409; Pre vs. 6 hpi) until 1 dpi (*p* = 0.0070; Pre vs. 1 dpi), after which CK values generally decreased (with the exception of DL666). The complete clinical chemistry data set can be found in Supplementary Data Sheet 1.

## Discussion

An ideal animal model of systemic MRSA infection should afford sufficient time between infection and a humane endpoint for the provision of antibiotics and/or other interventions (Chen et al., 2019). If the therapeutic window is too short, it can become practically challenging to test novel therapeutics before the onset of severe and irreversible illness. At the same time, a successful MRSA bacteremia model should accurately recapitulate clinical aspects of the condition, demonstrate activation of similar molecular pathways, and lead to the development of multiple organ dysfunction (MOD). In this study we present a stable, systemic NHP model of MRSA infection that produces a moderate disease state that is non-lethal for at least 8 days. This model serves as a complement to the well-established Hinshaw baboon model of *E. coli* bacterial sepsis (Taylor et al., 2012), which has served the research community for decades and elucidated many mechanisms of systemic bacterial infection.

### Establishment of MRSA bacteremia and systemic inflammation in the macaque

A short burst of bacteremia, likely resulting from the initial bolus of MRSA administered via IV, was detectable in all six core study animals 2 h after IV challenge. This “bolus effect” is well-documented in experimental models of sepsis (Lewis et al., 2016; Stortz et al., 2017) and—as expected—the spike at 2 hpi waned quickly as the host immune system began clearing the pathogen. However, at least four of the six infected macaques (DL606, DN361, DN508, and DN666) experienced robust secondary bacteremia peaks at or after 12 hpi, with one animal (DL606) only exhibiting peak bacteremia 8 dpi. This high inter-individual variation in the timing of secondary peak bacteremia suggests that the presence of MRSA bacteria in the blood at later time points results from productive MRSA infections established in various tissues and organs, as confirmed by histopathological analysis. While variable timing in the establishment of invasive infection can complicate analysis in this model, it was not an unexpected result. Baboon models of septic shock induced by intravenous infusion of *E. coli* exhibit a large range of bacteremia onset (Hinshaw et al., 1980; Hinshaw et al., 1981; Taylor et al., 2012), and a more recent macaque model of influenza/MRSA respiratory coinfection exhibited considerable variance in disease severity (Chertow et al., 2016; Kobayashi et al., 2013). Still, more sophisticated methods of bacterial challenge, such as implantation of bacteria-laden clots via intraperitoneal route or intravenous infusions over several hours, should be explored in NHPs to better mimic the bacteremia kinetics observed in most human cases of systemic MRSA infection, and to prevent endotoxic shock from high initial challenge doses.

Based upon the onset timing (∼18 hpi) of bacteremia in our 10^9^ CFU IV MRSA pilot animal, bacteremia and most other assay-based parameters were not assessed between 2 and 7 dpi. In hindsight, sampling should have been continued at least once daily for the duration of the study. Animal DN233 was euthanized at 2 dpi, before any robust infection was detected, though bacteremia in this animal was detectable in small amounts at the time of euthanasia. Animal DN362, which was not euthanized until 8 dpi, also did not exhibit robust bacteremia at the time points assessed. However, the high levels of CRP—a reliable marker of systemic inflammation—detected in this animal 7–8 dpi suggest that a bacteremia spike may have occurred in the days between 2 and 7 dpi. Elevated CRP was also detected in the two other animals euthanized 8 dpi. Considering that CRP has been shown to predict persistent MRSA bacteremia (Chung et al., 2021), these findings further support that a 10^9^ CFU IV dose of MRSA USA 300 is capable of establishing sublethal systemic infections in cynomolgus macaques. Future studies in this model will benefit from more frequent experimental time points after 2 dpi, and the inclusion of additional markers of inflammation, bacterial infection, and sepsis. Procalcitonin (PCT) is an established biomarker of blood infection, and is used to clinically to discriminate between bacterial and viral infections, and to inform antibiotic treatment regimens (Gregoriano et al., 2020). Elevations of both CRP and PCT were added to the updated definition of sepsis in 2003. Additionally, clinical studies of intensive “goal-directed” treatment of severe sepsis and septic shock used elevated lactate levels to guide therapy (Rivers et al., 2001), and obtaining a lactate level when monitoring patients at risk of developing sepsis has become standard practice. These and other options should be considered in study designs using the cynomolgus macaque to model systemic MRSA infection.

### Pilot study considerations and consequences

Despite the success of the pilot study in identifying a dose and route of MRSA USA 300 challenge capable of establishing a relatively stable systemic infection in cynomolgus macaques, some pilot findings were unexpected and warrant further investigation. The pilot animal that received a very high dose of MRSA (10^10^ CFU) via IP route did not exhibit bacteremia at any time point assessed (up to 4 dpi), despite the fact that abdominal sepsis has been established in baboons by implanting an *E. coli*-laden fibrin clot at a dose of 10^11^ CFU (Kinasewitz et al., 2000). It should be noted, however, that 3 of the 12 baboons in this study did not develop sepsis within 14 days and appeared clinically well.

Additionally, the pilot study was performed using animals immediately available to our research team, which precluded testing dose/route combinations on one animal from each sex; all pilot animals were female. With the intent to control our subsequent core study on the basis of sex, we screened 5 female and 5 male cynomolgus macaques for entry to the study. By our luck, however, all four animals that were excluded from the study (for presence of commensal *S. aureus* in nasal swabs) were female, resulting in a core study composition of five males and one female. This large difference in sex composition of pilot and core study groups may account for several study design limitations observed in retrospect. First, the speed of disease progression observed in the pilot animal that received the 10^9^ CFU IV dose selected for further study may have skewed our expectations regarding bacteremia kinetics for the core study, since this small female animal received 3.33 × 10^8^ CFU/kg, while, on average, the mostly male animals in the core study received ∼1.52 × 10^8^ CFU/kg. When selecting sampling time points for this study, we opted to focus on the first 48 h after infection, expecting most of the immunological and pathophysiological “action” to occur prior. Our findings in the core study, however, suggest that bacteremia as a result of invasive infection can occur up to 7–8 days post-challenge. Investigators using this model in the future may opt to dose the animals by body weight via a “low and slow” infusion, as in the Hinshaw baboon model (Taylor et al., 2012), or screen more animals to secure even sex ratios and appropriate statistical power for sex-based comparisons. Second, while sex is a neglected variable in infectious disease research, sex-dependent differences in incidence and severity of sepsis suggest that males are more susceptible to sepsis and septic shock than females (Lakbar et al., 2023). In our small core study, however, the lone female (DN508) experienced the earliest onset bacteremia, highest fever, and uniquely abnormal clinical chemistry. While this is insufficient for any speculation about sex-based differences in the cynomolgus macaque model of MRSA bacteremia, we encourage other researchers to approach this model with appropriate controls for sex and sex-associated differences.

### Clinical and hemodynamic features of MRSA infection in the macaque

Macaques in the core challenge study displayed hallmark clinical signs of bacteremia and sepsis, including elevated body temperature, weight loss, and hypotension. While only two animals reached clinical febrile thresholds for cynomolgus macaques (temperature above 36.5°C), rectal temperature measurements under sedation may underestimate true body temperature due to anesthetic-induced hypothermia (López et al., 2002). Future studies in this model may benefit from other thermometry techniques (e.g., implantable transponders) that can monitor body temperature continuously while the animal is conscious.

Body weight loss was observed in all core study animals, and persisted until study termination. In addition to providing a quantitative humane endpoint for studies in this model, body weight monitoring is economical to perform and can serve as an indicator of disease progression, which is rare in non-human primate models of invasive infections (2020). However, frequent sedations are known to contribute to decreased appetites in humans and non-human primates, which may confound the use of body weight as an indicator of disease progression and severity. Future MRSA bacteremia studies using the cynomolgus macaque may benefit from study designs that include less frequently sedated groups and other controls to help distinguish sedation-induced symptoms from those due to illness.

The vascular endothelium is a known target of *S. aureus* virulence, resulting in hemodynamic changes, especially failure to maintain proper blood pressure (Kwiecinski and Horswill, 2020). All animals except DN233 experienced decreased mean arterial pressure (MAP) within 24 hpi, and two of the three animals euthanized 8 dpi displayed blood pressure readings below normal clinical range. Considering that animal DN233 did not exhibit robust bacteremia before euthanasia at 2 dpi, and gram-positive cocci were not detected in its tissues, it is unsurprising that this animal presented with limited clinical signs of illness. Nevertheless, MAP estimation is an indirect measure of hemodynamic changes, and more accurate methods of hemodynamic monitoring can be achieved via use of an indwelling arterial catheter connected to an external pressure transducer. In this study, however, these methods were avoided since indwelling catheters are a complicating risk factor for the development of sepsis. Even without advanced methods, the cuff-based readings used in this study to assess blood pressure supported hemodynamic trends observed in human bloodstream infections and sepsis (Angus and van der Poll, 2013).

In future studies of MRSA bacteremia in the cynomolgus macaque, should monitoring extend beyond 8 days following challenge, these animals may require intensive care including IV fluids. To mitigate risks accompanied with sedation in septic animals, one potential approach for emergency medical intervention may include the use of tethering indwelling intravascular catheters to administer fluids or medications and the use of implanted transponders to measure vitals. However, these methods require surgical placement of devices, which may introduce complications like catheter-associated infections caused by non-target pathogens. Follow-up studies using this model should include non-fluid resuscitated control groups to ensure that any invasive management or monitoring methods do not introduce these complications.

### Hematological changes in MRSA-infected macaques

The observations of polychromasia (increased immature red blood cells), anisocytosis (variation in red blood cell size), poikilocytosis (variation in red blood cell shape), and hemolysis (breakdown of red blood cells) in MRSA-infected cynomolgus macaques suggest significant alterations in erythropoiesis and red blood cell integrity. These hematologic changes could be attributed to several factors associated with MRSA infection: (1) bacterial secretion of hemolysins, which are pore-forming toxins that lyse red blood cells (Chen et al., 2022), (2) reduction of vascular endothelial integrity, stimulating the release of immature or poorly-formed red blood cells to counteract vascular leakage (Kwiecinski and Horswill, 2020), and (3) liver and kidney damage, which is associated with poikilocytosis (Bandaru et al., 2025). It should be noted that many of these hematologic changes also accompany anemia, which could be the result of frequent blood collection and/or inappetence.

*S. aureus* employs various immune evasion strategies, including the manipulation of host coagulation pathways, to shield itself from immune attacks. In this study, platelet clumping was observed in most blood smears at 2 dpi and 8 dpi, fibrin strands were detected in 2 of 3 blood smears at 7 dpi, and a blood clot was identified at 2 dpi. These findings align with the known mechanisms of *S. aureus* immune evasion, as *S. aureus* infections are known to trigger coagulation cascades and promote the formation of fibrin shields around nidus structures, preventing host immune cells from accessing replicating *S. aureus* microcolonies (Kwiecinski and Horswill, 2020).

### Cytokine profile of MRSA infection in NHPs

Overall, the robust post-challenge expression of cytokines (IL-6, TNF-α, IFN-γ, G-CSF, and IL-1RA) and chemokines (IP-10, MCP-1, MIG, and MIP-1α) that we observed shared many similarities with the cytokine profiles of human patients with *S. aureus* bloodstream infections (Kim et al., 2020; Bergersen et al., 2025) and with bacterial sepsis generally (Angus and van der Poll, 2013).

IL-6 and TNF/TNF-α have been reported as early inflammatory markers of complicated *S. aureus* bacteremia (SAB) in clinical cases (Minejima et al., 2016; Kim et al., 2020), and both were induced shortly after MRSA challenge in the cynomolgus macaques in our core study. IL-6, a cytokine with pleiotropic activity, induces synthesis of CRP (Tanaka et al., 2014), a phenomenon we observed in 100% of our study animals euthanized 8 dpi. Across human patients with antibiotic-resolving and antibiotic-persistent SAB, Bergersen et al., report strongly elevated IP-10 (CXCL10) and MCP-1 (CCL2); patients in this study with antibiotic-persistent SAB showed increased expression of G-CSF, TNF-α, IL-6, and IFN-γ (Bergersen et al., 2025). It should be noted, however, that IL-8, IL-10, and sCD40L were also upregulated in SAB patients in this study, and our analysis of cynomolgus macaque cytokine profiles showed either no upward trend in cytokine concentration (as with IL-8), or did not detect these cytokines above the limit of detection in > 50% of animals (as with IL-10 and sCD40L). We did observe elevated IL-10 values in a single animal (DL606) at 0, 2, and 24 hpi. Conversely, IL-1RA was not detected in sufficient samples for inclusion in the Bergersen study, but was observed to spike dramatically in all six of our MRSA-challenged core study macaques. To date, no comparable cytokine profile information exists for MIG, which we also observed to spike shortly after MRSA challenge. IL-1β, IL-5, and IL-7, which we did not detect within range in our Luminex^®^ assay, have been implicated in the host response of *S. aureus* infections in murine models (Li et al., 2021), but were not reported by Bergersen et al., or any other clinical studies. This lends further support to the idea that development of NHP models of MRSA bacteremia is an important step in translational research for the development MRSA vaccines and therapeutics.

One known feature of sepsis is an immunosuppressive response which occurs simultaneously and paradoxically with the inflammatory response. It is characterized by the release of anti-inflammatory cytokines, death of leukocytes, T-cell exhaustion, and excessive production of immunomodulatory cells (Liu et al., 2022). While IL-10, a major anti-inflammatory cytokine observed in both clinical and animal model studies of *S. aureus* bacteremia (Bergersen et al., 2025; Leech et al., 2017), was detected in only one animal (DL606), we did observe a significant spike in IL-1RA, a natural antagonist that blocks the pro-inflammatory activity of IL-1β and IL-1α by competitively binding to the IL-1 receptors. This may account for the lack of detectable IL-1β in our study, and could also be an indirect cause of the relatively stable levels of IL-8, as IL-1RA has been demonstrated to inhibit IL-1-induced IL-8 production via inhibition of p38 mitogen-activated protein kinase and NF-κB pathways (Garat and Arend, 2003). Additionally, elevated IL-10 is largely observed in patients with more severe disease and higher mortality (Rose et al., 2012; Kim et al., 2020), which are undesirable outcomes in a sublethal NHP model of MRSA bacteremia. Other cytokines with anti-inflammatory activity, including G-CSF and IL-1RA, were observed to spike simultaneously with inflammatory cytokines in the sera of core study animals.

Interestingly, we observed an increase in IFN-γ in animal DN508 orders of magnitude greater than in other animals at 6 hpi. While IFN-γ is generally associated with innate and adaptive immune responses to viral pathogens, it is reported that *S. aureus* superantigens can elicit pathologic levels of IFN-γ, which enhance disease severity and bacterial burden (Tuffs et al., 2022). Animal DN508 displayed gram-positive cocci in both the spleen and heart just 48 h after MRSA challenge, and presented with the earliest peak bacteremia at 12 hpi. It is also possible that an unrelated subclinical inflammatory process was already taking place in this animal prior to MRSA challenge, predisposing DN508 to more severe MRSA infection. We encourage future researchers using this model to evaluate local cytokine and chemokine responses in addition to assaying for circulating molecular signals. Still, the cytokine activity captured in this study demonstrates that an inflammatory process was detectable post-infection and could be monitored beyond 24 h. These findings reinforce the cynomolgus macaque as a promising animal model for future MRSA studies.

### Neutrophils and associated cytokines

MRSA-infected cynomolgus macaques exhibited elevated white blood cell counts and mild thrombocytopenia concordant with human clinical cases. A sharp increase in total white blood cells (leukocytosis) within hours of infection, primarily to an increase in circulating neutrophils, suggests an acute inflammatory response to MRSA challenge. In response to bacterial infections, pro-inflammatory cytokines including IL-8, IL-6, TNF-α, and IL-1β are released, triggering neutrophil recruitment and new neutrophil production via G-CSF (Tsioumpekou et al., 2023). While a significant spike in TNF-α and IL-6 was observed in our study animals 6 hpi, circulating IL-1β could not be detected, and IL-8 levels appeared largely unchanged. Considering the significant spike in G-CSF at 6 hpi, it is possible that these unexpected results were the result of low assay sensitivity (in the case of IL-1β) or model-specific differences (in the case of IL-8). Elevated IL-8 has been observed in human keratinocytes infected with MRSA USA300 (Ngo et al., 2022), and in baboon models of gram-negative sepsis (Creasey et al., 1991), so additional studies are needed to determine if or how IL-1β and IL-8 are involved in the innate immune response to MRSA in the cynomolgus macaque.

It is also of interest that while neutrophil counts remained elevated in serum far above baseline throughout the study, there was a distinct recovery toward normal clinical levels beginning at 12 hpi and persisting though at least 2 dpi. This is in conflict with the extended elevation of circulating neutrophils observed in many clinical cases. In our study, depletion of circulating neutrophils after 12 hpi may have been due to large-scale recruitment of neutrophils to multifocal sites of bacterial infection that could not be entirely compensated for by scaling up neutrophil production and/or maturation. While neutrophil recruitment occurs in the same way in humans, natural MRSA infections generally do not begin with establishment of numerous bacterial niduses across all tissues and organs as a consequence of extremely high bacteremia. The circulating neutrophil depletion we observed shortly after experimental infection with MRSA may be another component of the “bolus effect”, in which many elements of the innate immune system are exhausted by the initial onslaught of bacteria, leaving the host less able to defend against secondary infections.

While our flow cytometry immunophenotyping panel was designed to evaluate total granulocytes, neutrophils, and neutrophil activation/migration, we could not identify a robust neutrophil population via flow cytometry, even at time points exhibiting extremely high neutrophil counts as determined by CBC. This issue was likely to due to incorrect gating parameters set for neutrophil populations, or an issue with neutrophil-related antibody stains. It is also possible that this issue resulted from the use of a “live gate,” in which side scatter and forward scatter properties were used to indirectly determine cell viability. However, prior optimization for this assay demonstrated minimal non-viable cells in whole blood during acquisition. While use of a viability dye is strongly preferred, the “live gate” approach is a known viability determination alternative in cases where channels are in extremely high demand. Future investigators employing flow cytometry immunophenotyping in this model should attempt a different granulocyte gating strategy, optimize the method for neutrophil phenotyping, and employ a viability dye. Changes in neutrophil phenotypes after bacterial challenge could also be investigated through established transcriptomics/proteomics methods and migration assays.

### Monocytes and associated cytokines

In our sublethal model, the robust host cytokine and chemokine response observed within 6 h of infection corresponded with a drastic reduction in circulating classical monocytic cells, which represent up to 90% of monocytes under homeostatic conditions. This loss could be attributed to the differentiation of classical monocytes into macrophages and dendritic cells, or a possible increase in the marginal pool (van Furth and Sluiter, 1986). Conversely, a significant increase in the intermediate monocyte subset was induced 6 hpi, similar to trends observed in patients with acute sepsis (Hortová-Kohoutková et al., 2020). Under steady state conditions, 1% of classical monocytes are believed to differentiate into intermediate, and subsequently non-classical monocytes in the periphery (Patel et al., 2017). It is unclear whether systemic *S. aureus* infection and sepsis can dysregulate this process, possibly inducing a greater proportion of classical monocytes to undergo differentiation when in a diseased state (Karauzum and Datta, 2017).

### T cells, B cells, and NK cells

While *S. aureus* superantigens have been shown to be potent T and B cell activators, experiments in murine models suggest that T and B cells are largely dispensable in early *S. aureus* infection (von Köckritz-Blickwede et al., 2008; Gjertsson et al., 2000; Bröker et al., 2016). In this study, minimal proliferative change was noted in peripheral T cells (both CD4 + and CD8 +) and B cells through 8 dpi. This result was not unexpected, as robust T and B cell proliferation is generally not observed in macaques until at least 2 weeks after infection or exposure to immunogen. Still, a significant gap in the field is the lack of an effective vaccine against *S. aureus*, which will require the successful manipulation of adaptive immunity. Though preclinical vaccine studies in mice initially produced promising results, subsequent clinical trials have failed to show efficacy when targeting capsular polysaccharides, cell surface antigens, or monoclonal antibodies (Kim et al., 2014; Clegg et al., 2021). In the interest of improving our NHP model’s translational potential for vaccine development, it will be necessary to further investigate adaptive immune cell behavior in MRSA-infected cynomolgus macaques.

In contrast to the limited fluctuations in T and B cell populations, changes to the peripheral NK cell population after MRSA challenge were significant. NK cells are directly activated by *S. aureus in vitro*, and activated NK cells are heavily recruited from circulation to sites of *S. aureus* infection (Small et al., 2008; Torcellan et al., 2024). In our study, NK cells were quickly depleted from circulation by 2 dpi, and remained well below baseline until at least 12 hpi. With this information, we expect that future experiments investigating the role of NK cells in *S. aureus* infections will be well-supported by this model.

### Tissue pathology and markers of organ dysfunction

Once MRSA enters the bloodstream, it can infect nearly any tissue or organ type, causing dysfunction due to inflammation and necrosis around bacterial niduses (Tong et al., 2015). As early as 2 dpi, we observed gram-positive cocci by histopathologic microscopy in the spleen and heart of animal DN508, accompanied by neutrophilic inflammation and infarction/necrosis. These necrotic foci were visible on the spleen by gross pathology. This animal also displayed one of the earliest peaks in CK, a serum biochemistry marker historically used to identify damage to the myocardium (Bodor, 2016). Interestingly, this animal and the other animals euthanized at 2 dpi also exhibited microscopic and biochemical indicators of liver and kidney dysfunction, despite the absence of bacterial cocci in the histological sections of these tissues collected at this time point. While it is certainly possible that limited histological sections of these organs failed to capture bacterial niduses already present (gross pathology of the kidney of animal DN666 showed necrotic lesions), systemic inflammation in conjunction with immune suppression is known to result in secondary damage of neighboring tissues, rendering the host further susceptible to the pathogen (Angus and van der Poll, 2013). Moreover, Kupffer cell hyperplasia and/or hypertrophy was observed in the livers of all animals assessed at 2 dpi; these liver-specific macrophages are a first line of defense against bacterial infection, but can also serve as “Trojan horses” for *S. aureus*, turning the liver into a source of metastatic infection (Surewaard et al., 2016; Pollitt et al., 2018; Jorch et al., 2019). Even healthy activation of Kupffer cells in response to bacteremia results in the expression of inflammatory cytokines, including TNF-α and IL-6, which could account for the inflammation observed in the liver at early time points, as well as the sharp increase of both these cytokines observed in serological analyses.

By 8 dpi, histological findings were widespread, and gram-positive cocci were observed in lung, kidney, spleen, and heart. The spleen seemed particularly susceptible to invasive MRSA infection, with all three animals euthanized 8 dpi displaying gram-positive cocci in splenic histological sections, accompanied by splenomegaly in gross examination. Notably, no staphylococci were observed in the liver of these animals at this later time point, though neutrophilic inflammation and Kupffer cell hyperplasia/hypertrophy were still pervasive, and one animal (DN361) presented with moderate enlargement of the organ at gross necropsy. Serological markers of liver damage and dysfunction, namely AST and ALT, were elevated between 6 and 1 dpi, after which they returned largely to baseline. This brief, early spike further supports the idea that liver function was compromised not by progressive MRSA infection of the liver tissue, but possibly by inflammation caused by the immune system’s initial clearance of the bacterial challenge. Interestingly, while all six animals displayed minimal to moderate microscopic changes in the liver, only animal DN508 displayed notably elevated total TBIL, another marker of hepatic dysfunction. TBIL is produced during the breakdown of hemoglobin, which is transported to tissues via erythrocytes. Unconjugated TBIL is transported through the blood to the liver, where it is taken up by hepatocytes and conjugated (Bain and Latimer, 2011). The observed increase in TBIL at 6 hpi may be due to hemolysis, which was observed in the blood smears of all study animal smear preparations but at a higher index value in animal DN508 at 6 hpi. Hemolysins are an important virulence factor for MRSA and destruction of red blood cells is an expected outcome of MRSA bacteremia (Vandenesch et al., 2012). GGT levels remained largely within normal clinical range, with the exception of animal DL606, which displayed elevated GGT at 8 dpi. This finding supports the severe liver pathology observed in this animal compared to other animals evaluated at this time point.

All but one animal in this study (DN666, euthanized 2 dpi) displayed gross kidney lesions at necropsy. The animals exhibiting gross kidney lesions also presented with varying degrees of inflammation, tubular degeneration, and necrosis throughout the tissue at the microscopic scale. At 2 dpi, there were no bacteria present on the Gram and H&E-stained kidney sections. However, abundant gram-positive cocci accompanied by acute microthrombi were evident in one animal (DL606) by 8 dpi, and all three animals euthanized at this time point demonstrated characteristics of chronic inflammatory changes and renal necrosis. The trends observed in serum protein ratios support a decrease in kidney function (decreasing ALB/GLOB) due to organ damage (increasing BUN/CRE) (Bain and Latimer, 2011). In humans, MRSA carries the potential to cause glomerulonephritis due to the formation of immune complexes at the glomeruli, the primary filtering structures of the kidneys (Koyama et al., 1995).

Remarkable cardiac lesions were observed in histopathologic analyses of MRSA-challenged animals at both 2 and 8 dpi. Interestingly, the three animals euthanized at 2 dpi presented more severe microscopic heart lesions (neutrophilic inflammation and myocardial necrosis) compared to the animals euthanized at 8 dpi. All animals experienced abnormal elevations of serum creatine kinase (CK) post-challenge. CK is a highly sensitive serum enzyme indicator of striated muscle damage (Hall et al., 2011) and is a historical diagnostic marker of myocardial injury (Bodor, 2016). Significant CK elevations were observed in study animals as early as 6 hpi, with all animals displaying significantly elevated CK levels of from 12 to 1 dpi. These increases in CK also were found to coincide with significant spikes of serum AST, supporting the occurrence of a necrotizing muscle injury (Bain and Latimer, 2011). These cardiac findings mirror the lesions defining MRSA endocarditis seen in human patients (Kaushik et al., 2024), and satisfy the Modified Duke Criteria for confirmatory diagnostic methods of infective endocarditis, including microorganisms demonstrated by culture or histological examination of a vegetative lesion, the presence of embolized vegetation, and/or an accumulation of white blood cells, particularly neutrophils, in the tissue (Baddour et al., 2015).

### Additional study limitations and future directions

To establish a true baseline for systemic MRSA infection in the cynomolgus macaque, we originally selected ten animals (5 female, 5 male) to be challenged without fluid resuscitation or antibiotic treatment in the core study. However, four animals, all female, were excluded during the pre-study screening process, and could not be replaced within the time frame allotted for our study. While a sample size of six animals is modest compared to equivalent small animal studies, it provided sufficient power to observe a variety of physiological, hematological, immunological, and histopathological phenomena while limiting the total number of animals used, in alignment with current recommendations for humane care and use of primate research species. Still, future studies using the cynomolgus macaque model of systemic MRSA infection will benefit from larger groups sizes, controls for sex and other biological variables, and the inclusion of antibiotic-treated and/or fluid resuscitated control groups, as is encouraged by investigators in the field of sepsis research (Chen et al., 2019).

Based on the speed of disease progression observed in the pilot animal that received the 10^9^ CFU MRSA challenge, as well as prior sepsis studies in the Hinshaw baboon model, we expected to observe most of the immunological and physiological events associated with MRSA infection prior to the 48 hpi time point. However, due to temporal variation in the establishment of bacteremia and inter-individual differences in tissue tropism, the lack of sampling time points between 2 and 7 dpi left a notable gap in our study. Future studies using this model should also include more frequent sampling between 2 and 7 dpi to capture an uninterrupted view of MRSA infection. Additionally, longitudinal tissue bacterial load quantification would further elucidate organ-specific tropism and infection kinetics.

## Conclusion

In summary, we demonstrate a relatively stable systemic MRSA infection in the cynomolgus macaque that recapitulates key clinical, immunological, and histopathological features of systemic human MRSA infections. The route, dose, and strain of MRSA challenge assessed in this study provides an extended therapeutic window, enabling longitudinal assessment of host-pathogen interactions and evaluation of novel therapeutics under controlled experimental conditions. Our hope is that future studies in this non-human primate model will facilitate the development of targeted interventions against MRSA, bridging the gap between rodent studies and clinical trials.

## Data Availability

The original contributions presented in the study are included in the article/Supplementary material, further inquiries can be directed to the corresponding author.
