# Supplementary figures and images for: A stable systemic infection of methicillin-resistant Staphylococcus aureus (MRSA) in cynomolgus macaques produces extended window for therapeutic intervention
Source: Front Microbiol. 2025 Jul 14;16:1601381. doi: 10.3389/fmicb.2025.1601381 (PMC12322839; doi:10.3389/fmicb.2025.1601381)

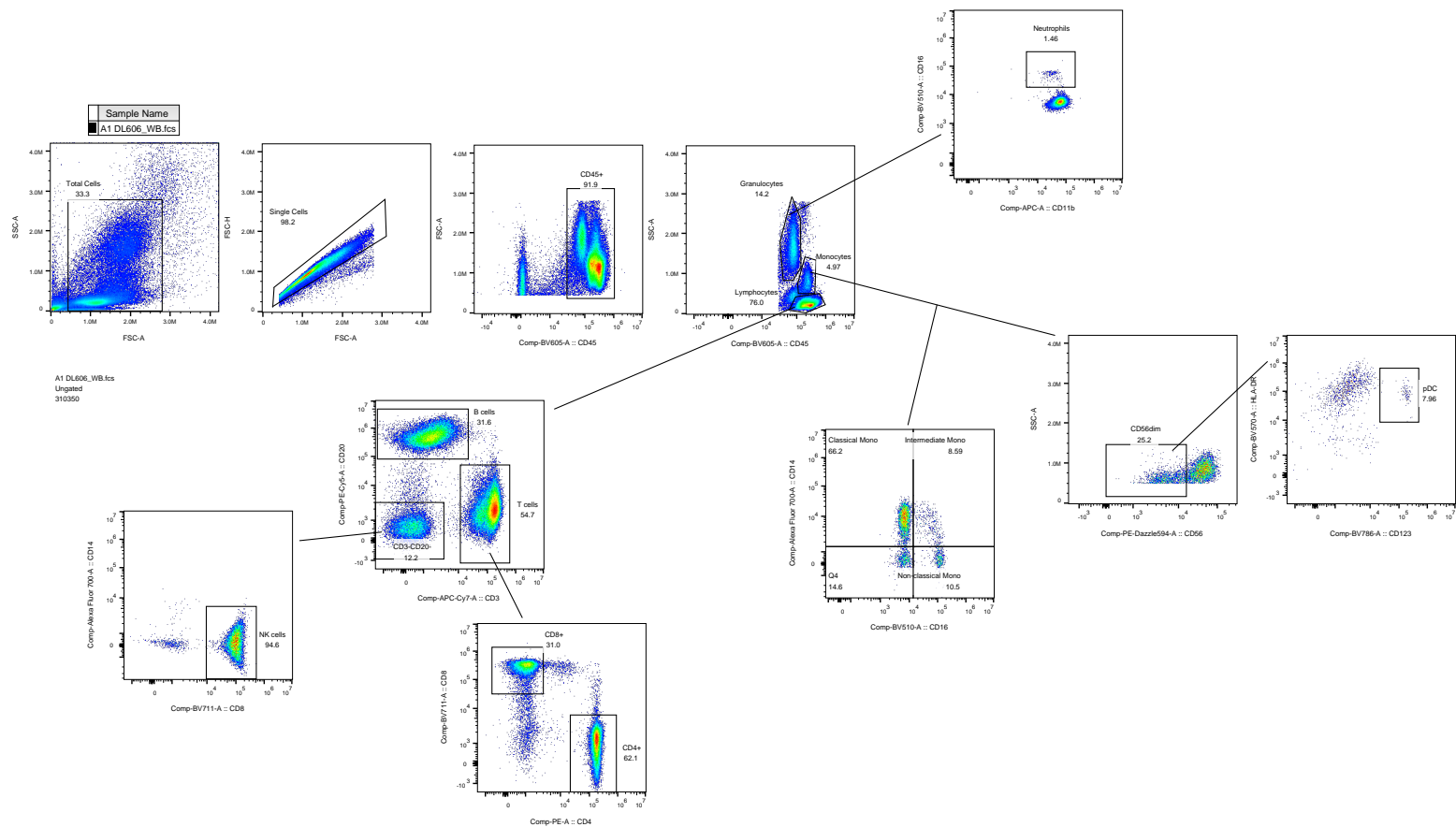

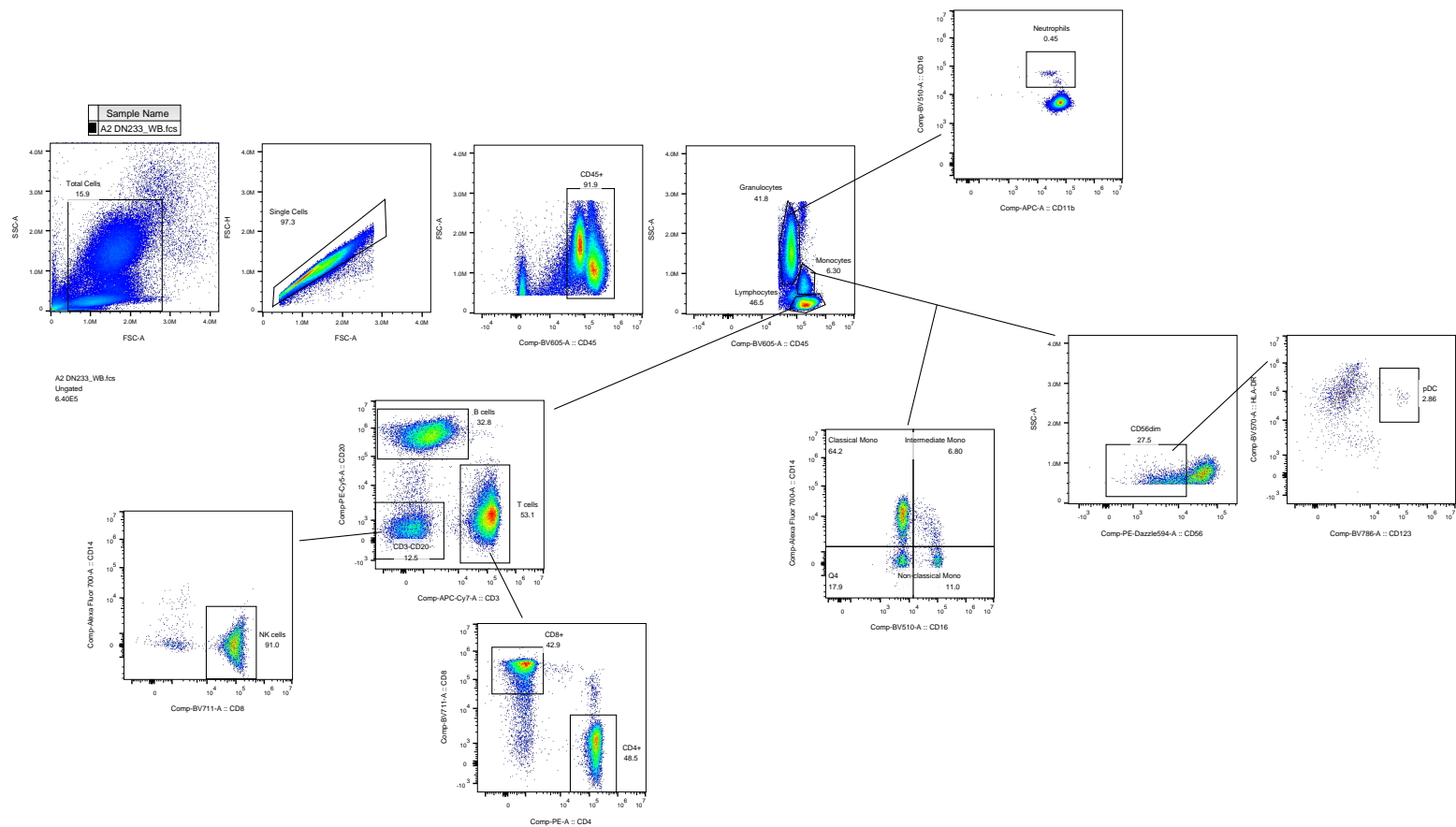

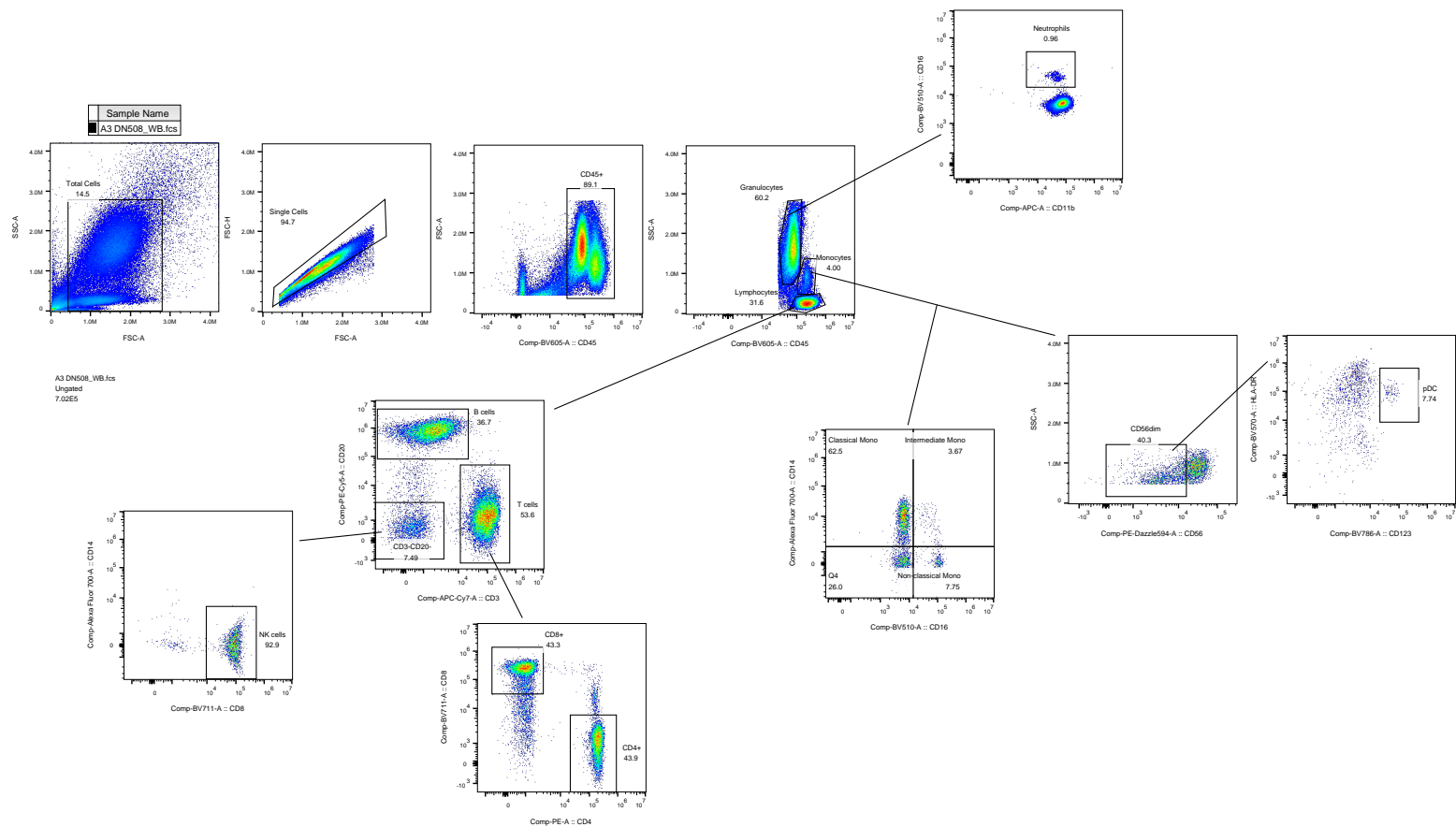

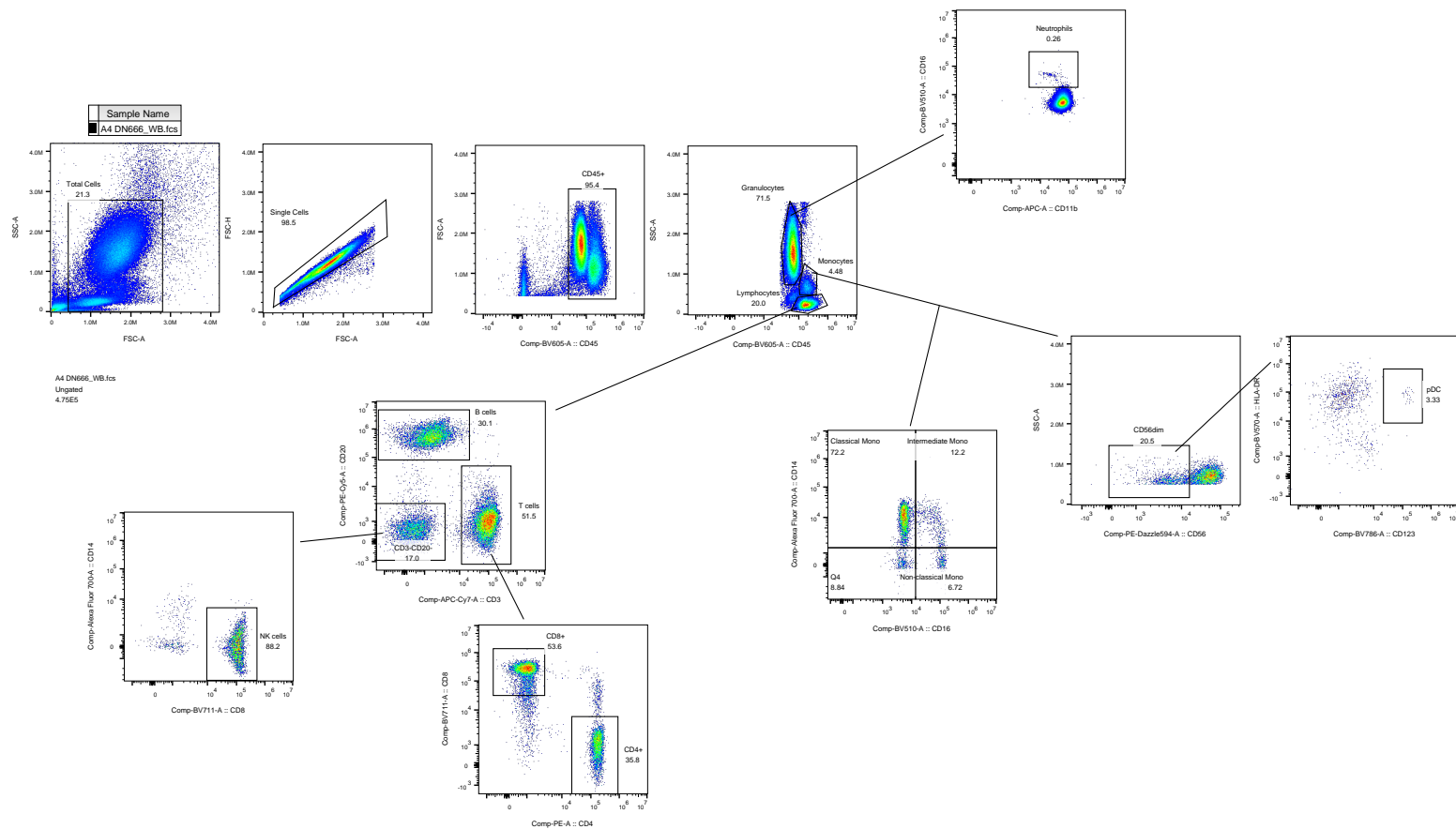

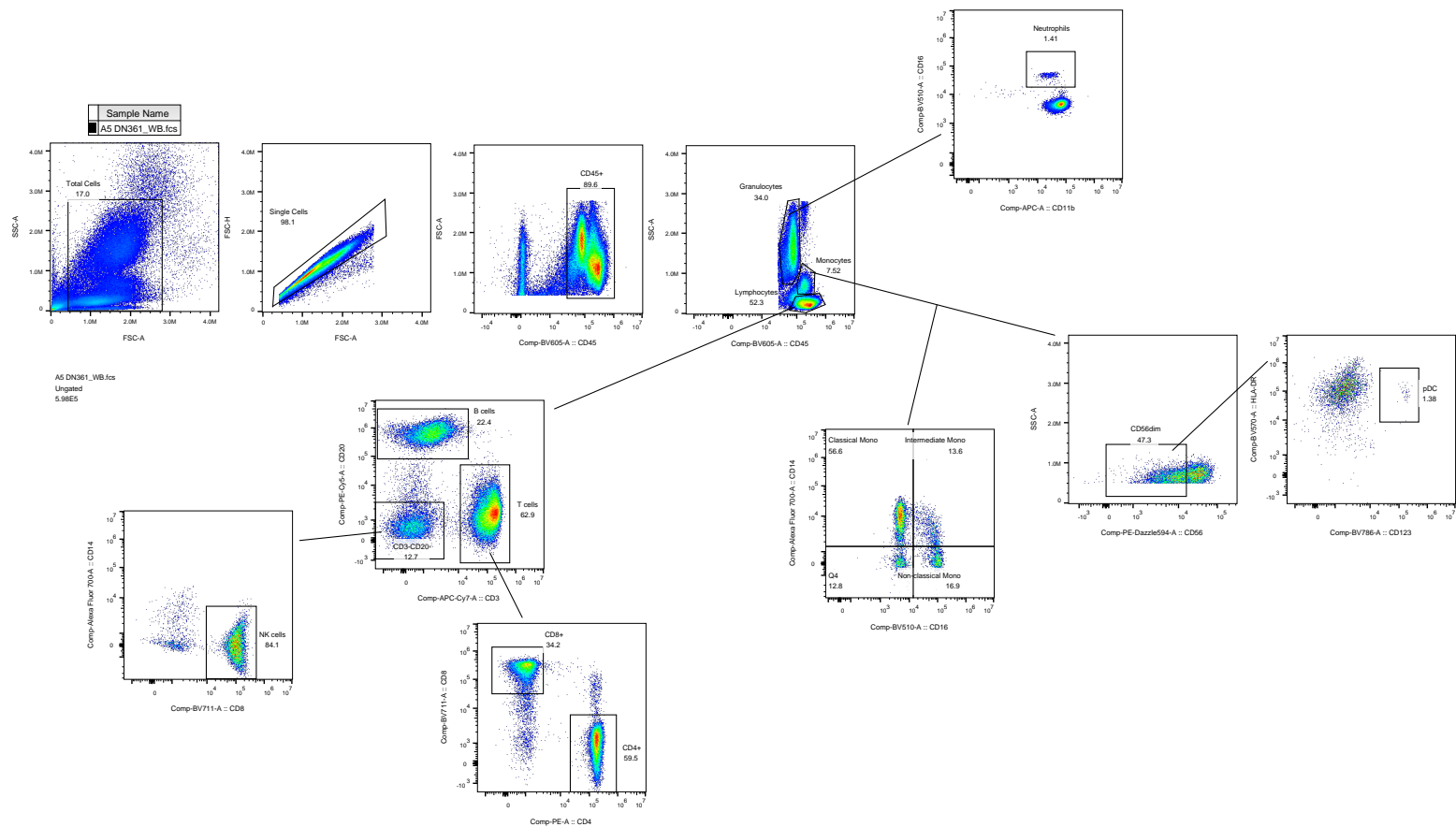

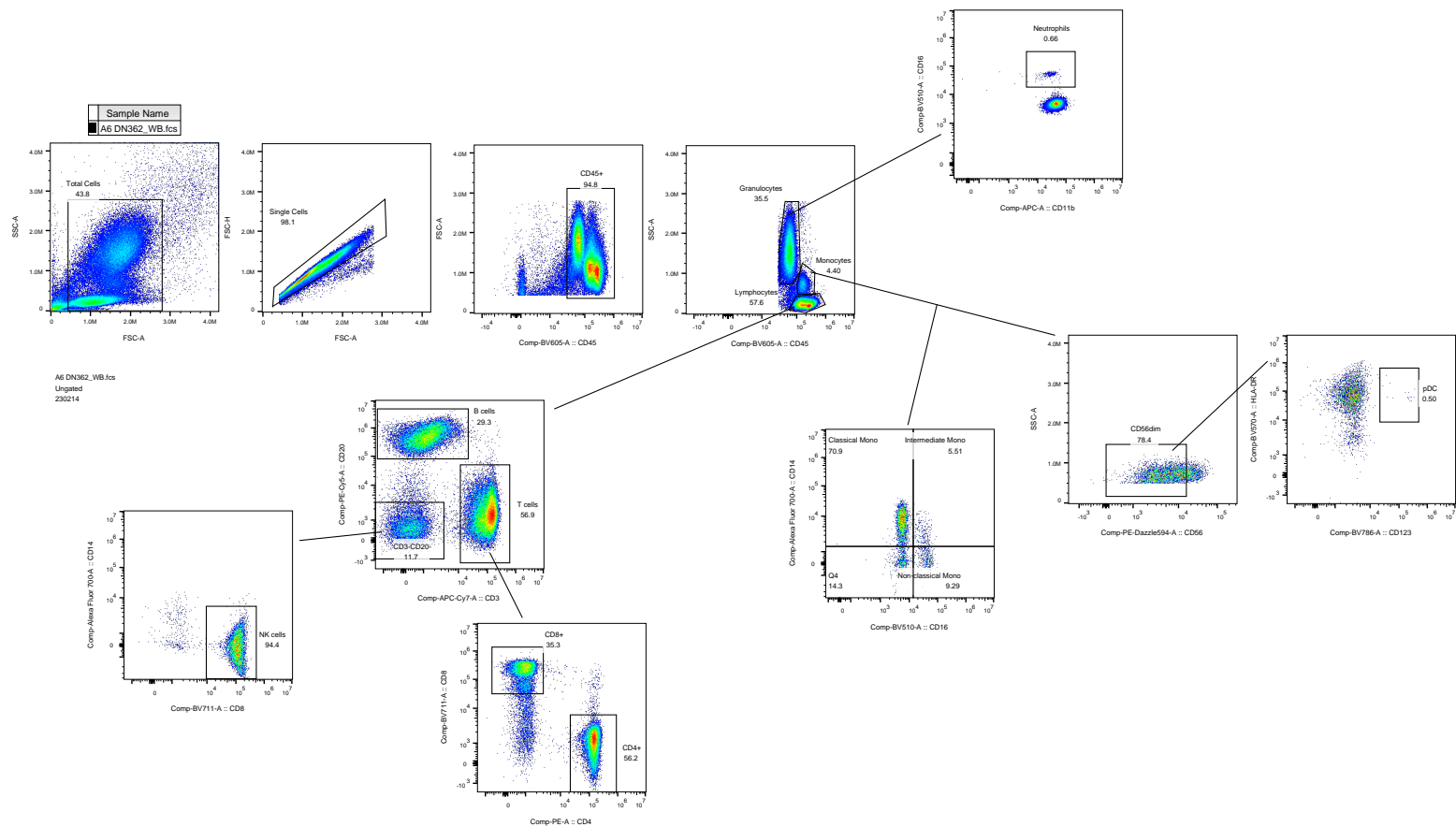

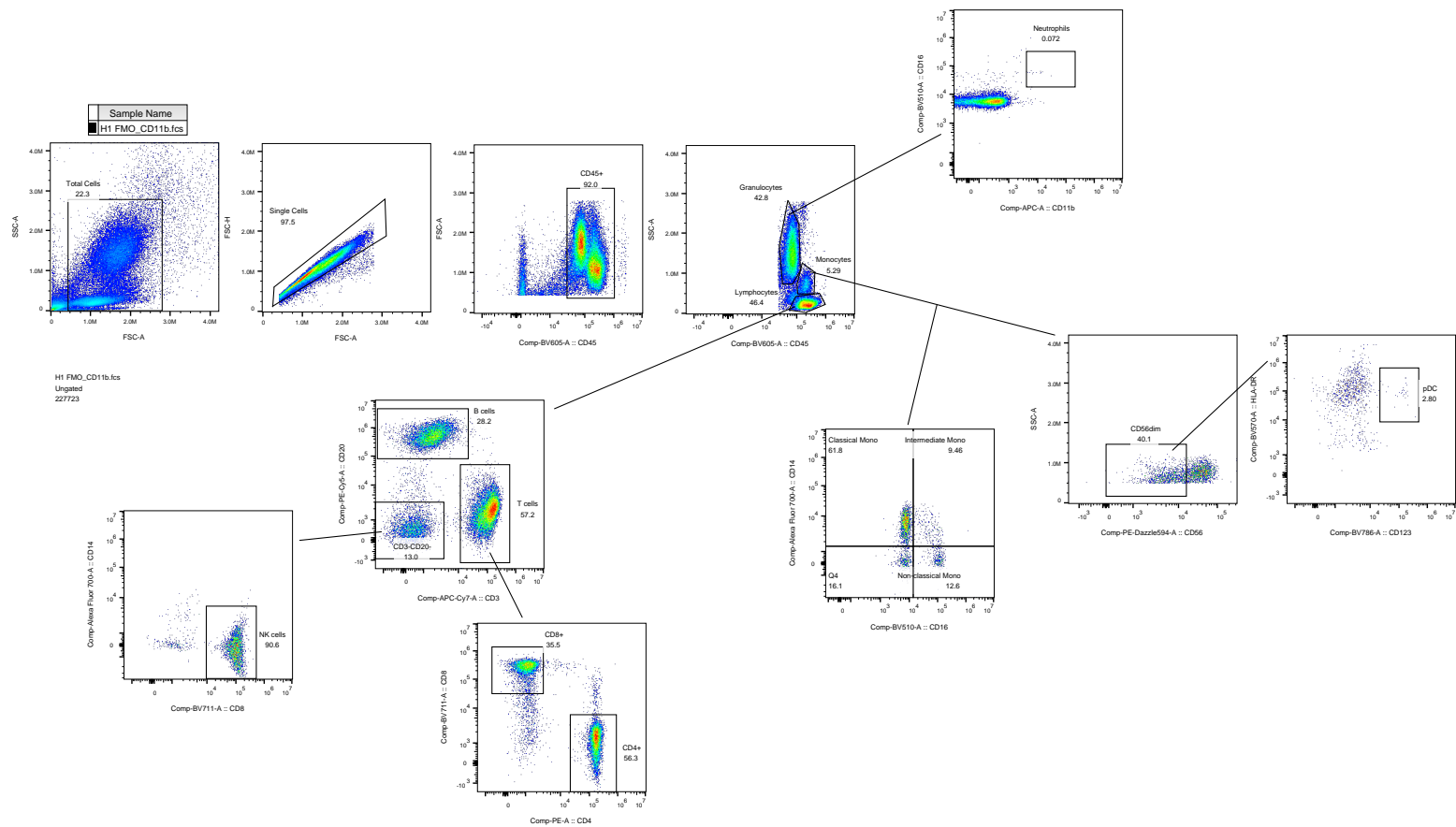

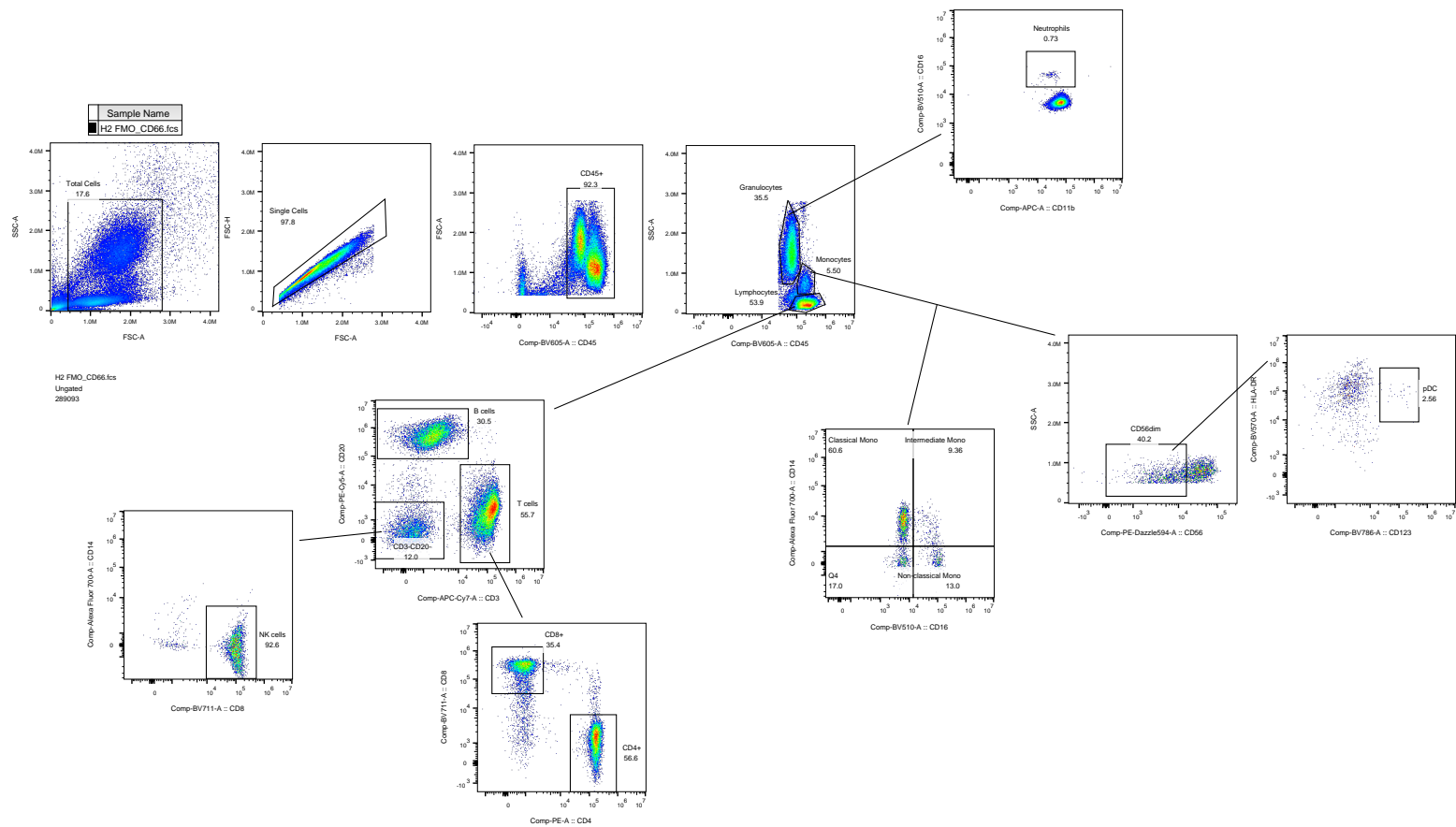

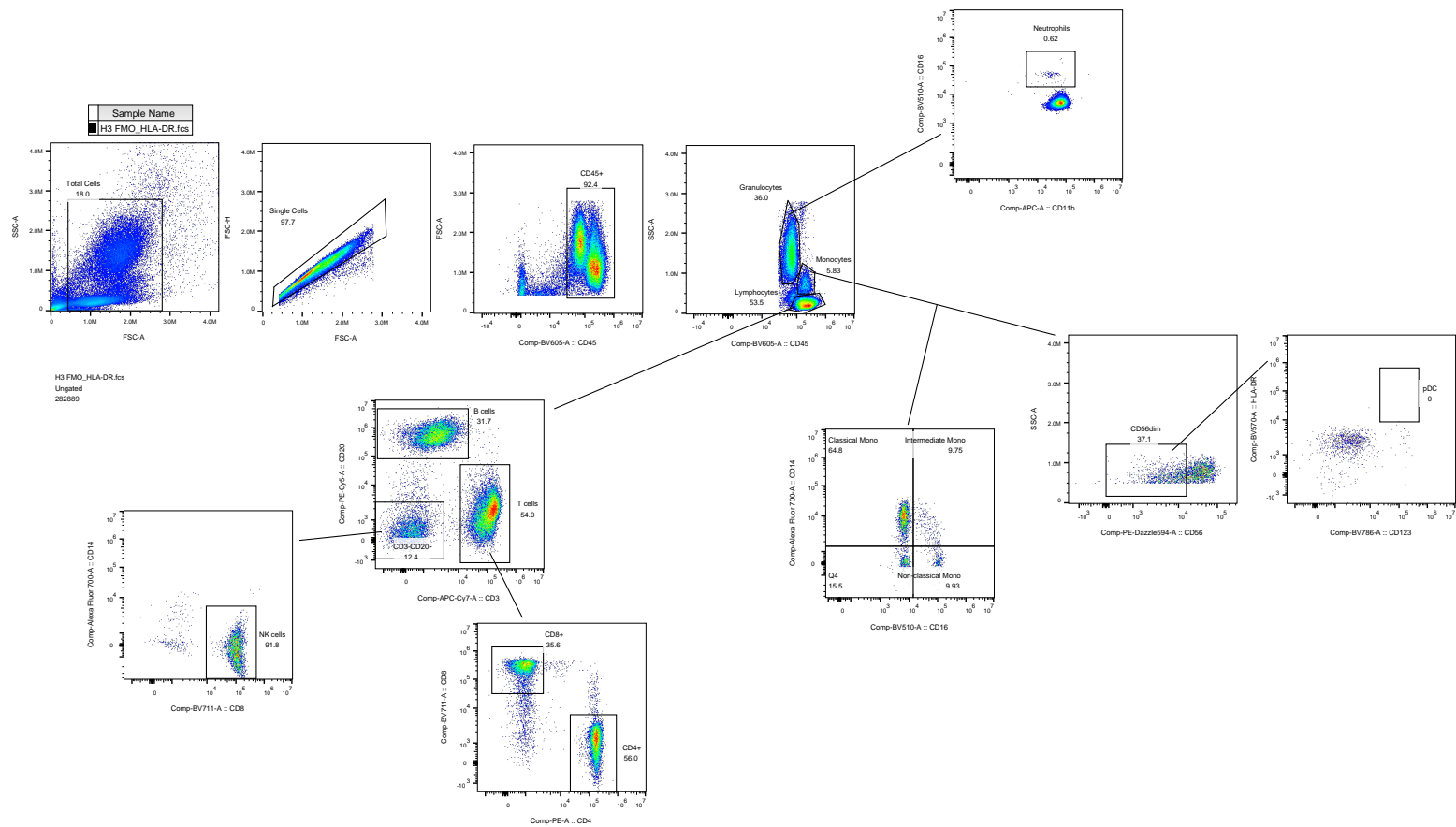

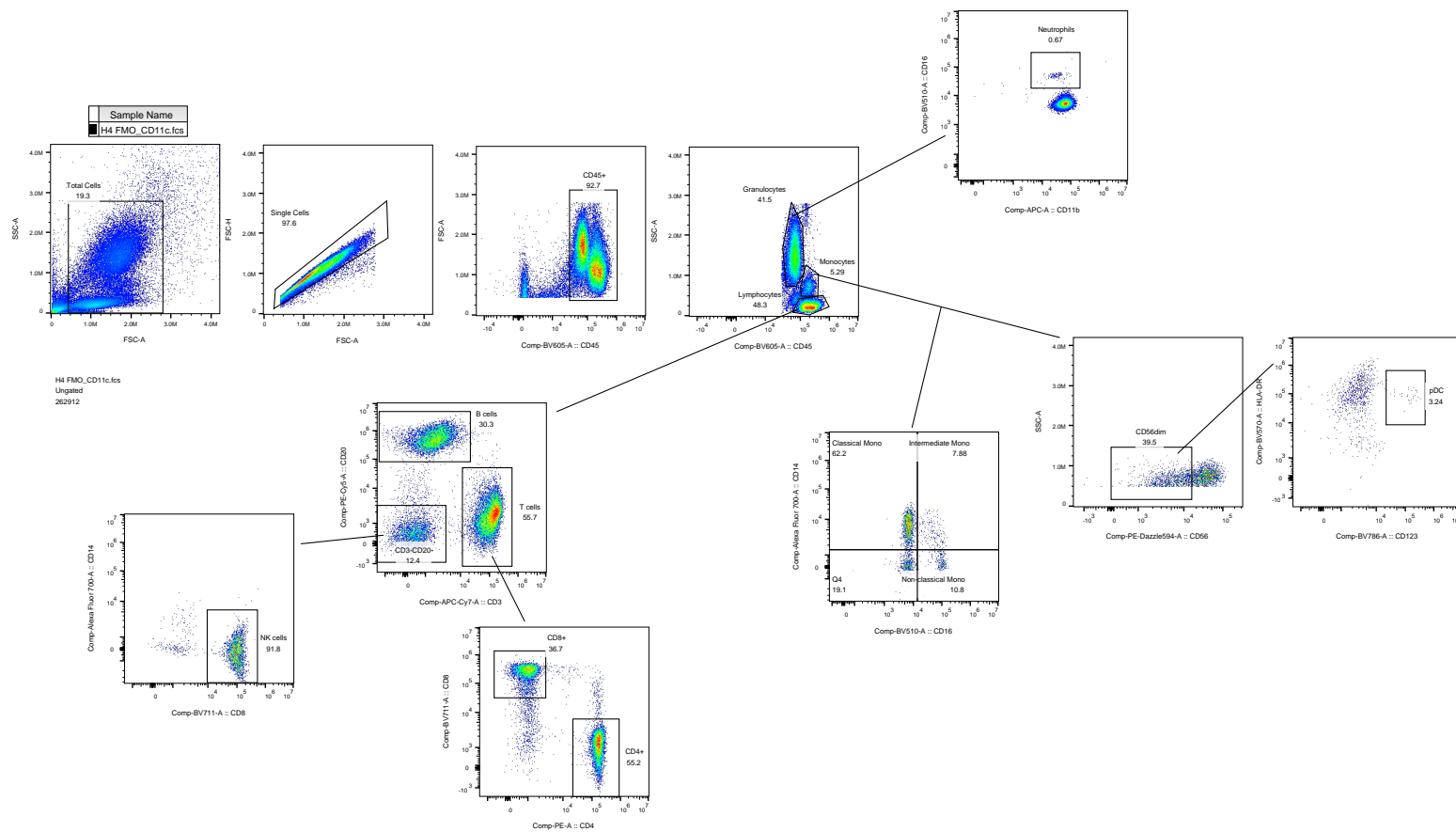

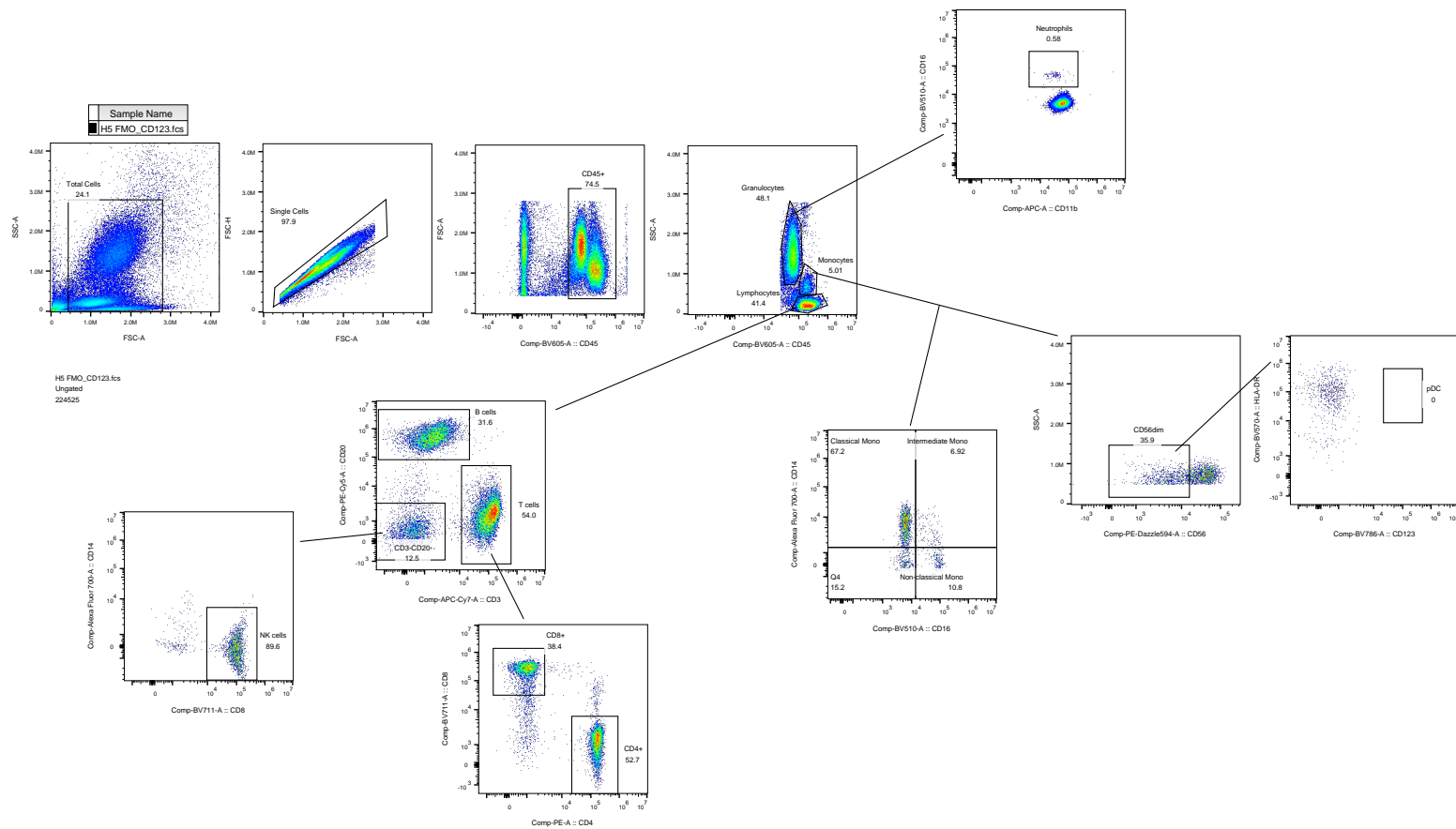

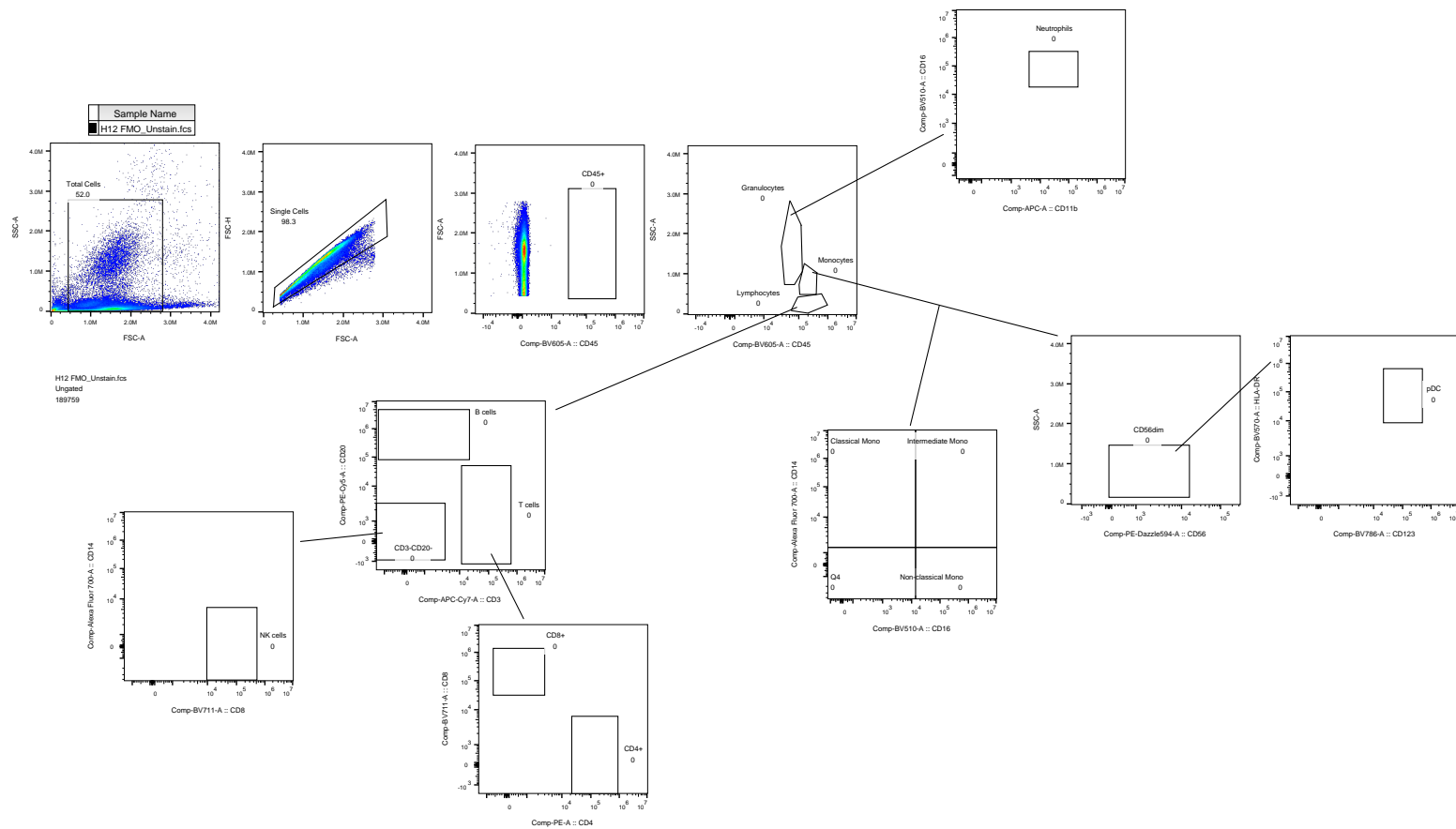

Supplement: Supplementary file 1 [file Presentation_1.pdf]

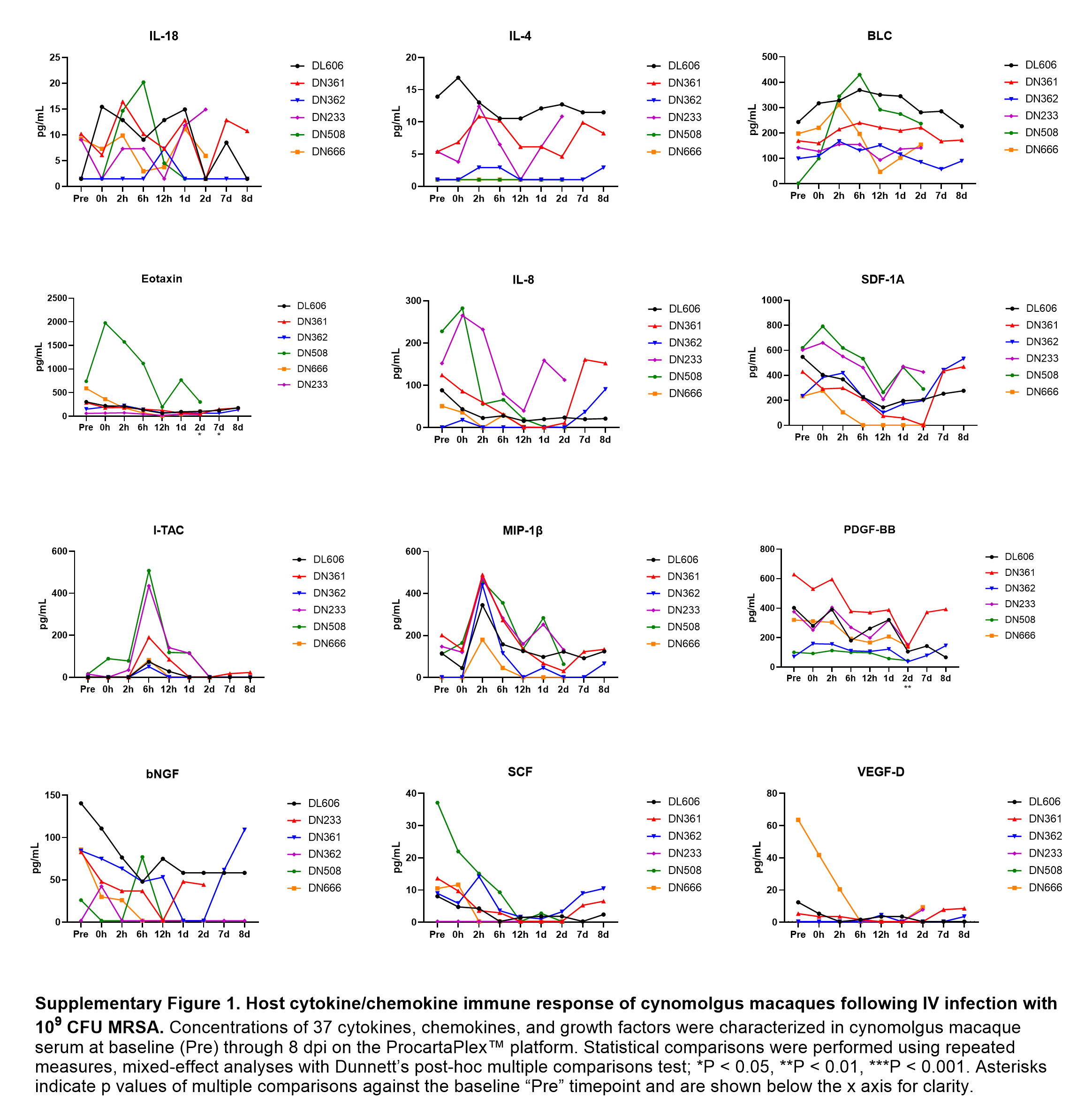

Supplement: Supplementary file 5 [file Image_1.tif]

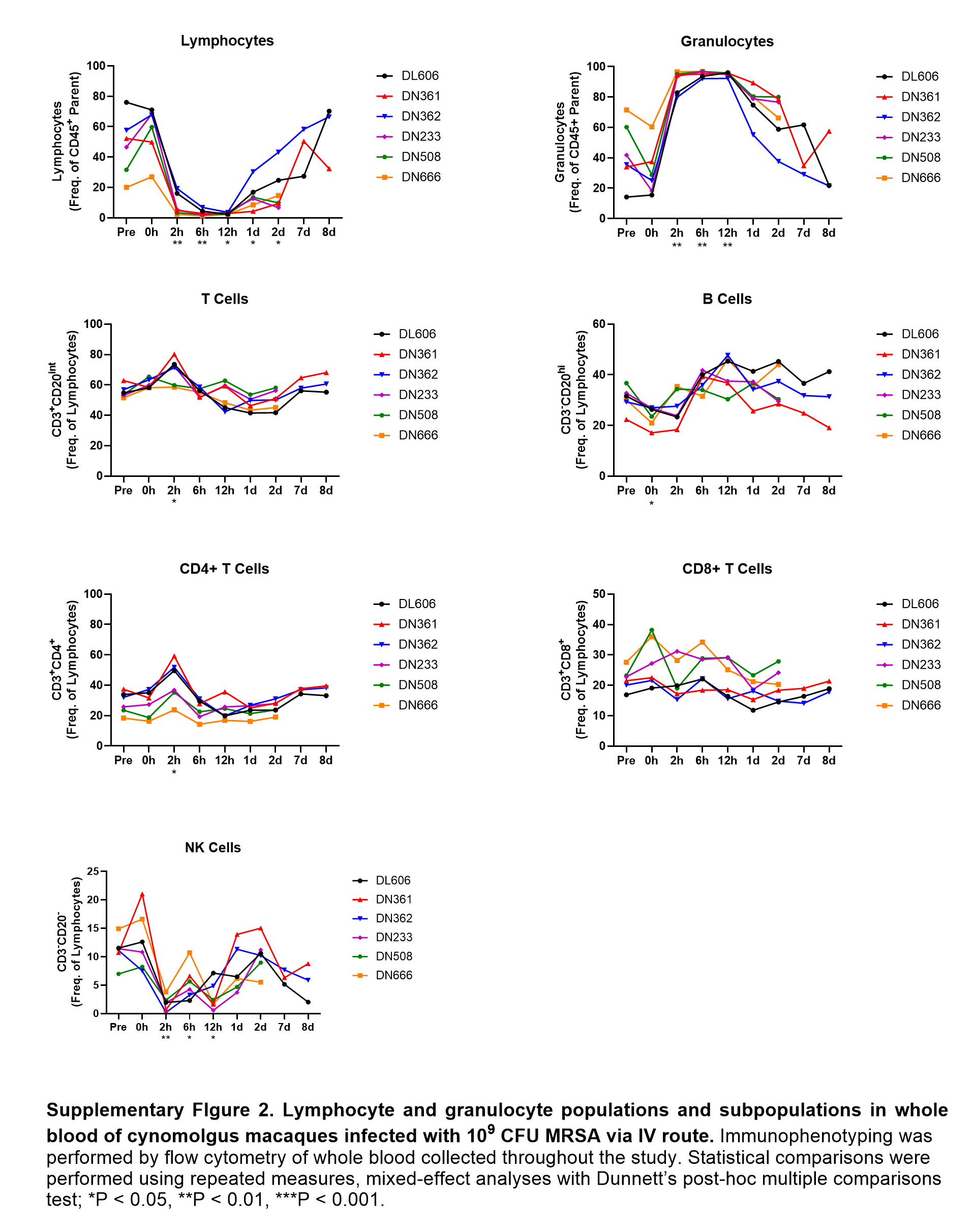

Supplement: Supplementary file 6 [file Image_2.tif]

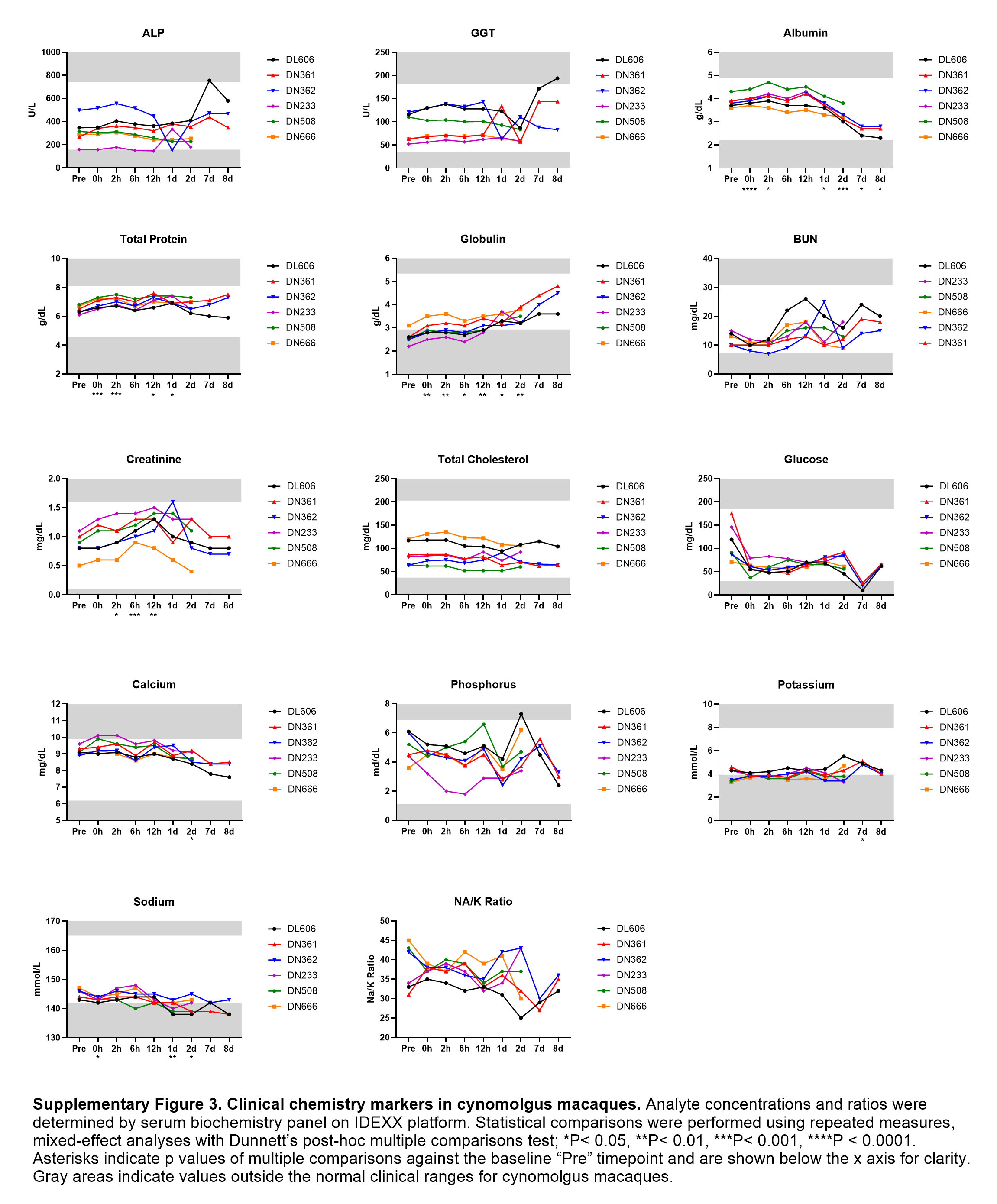

Supplement: Supplementary file 7 [file Image_3.tif]
